# A meta-analysis of the pooled impact of *CYP7A1* single nucleotide polymorphisms on serum lipid responses to statins

**DOI:** 10.3389/fgene.2023.1199549

**Published:** 2023-06-09

**Authors:** Megan Yu Cai Lim, Jia Rong Tee, Wai-Ping Yau, Han Kiat Ho

**Affiliations:** ^1^ Department of Pharmacy, Faculty of Science, National University of Singapore, Singapore, Singapore; ^2^ Department of Mathematics, Faculty of Science, National University of Singapore, Singapore, Singapore

**Keywords:** pharmacogenomics, CYP7A1, single nucleotide polymorphisms, statins, lipid response

## Abstract

**Background and Aims**: Various publications suggested that there is an association between *CYP7A1* single nucleotide polymorphisms (SNP) and a reduced response to statin therapy, but the results were inconsistent. This study aimed to collectively review these publications to appraise the effect of statins on cholesterol control in carriers of *CYP7A1* variant alleles.

**Methods:** PUBMED, Cochrane and EMBASE were searched systematically to identify reported studies on the lipid responses to statin treatment between carriers of the variant allele *versus* the non-variant allele of *CYP7A1* SNPs. The change from baseline in lipid responses for all included studies were calculated using weighted mean differences (WMD) (with 95% confidence interval (CI)). A meta-analysis was conducted to pool results using either the random-effects model or the fixed effects model.

**Results:** A total of 6 publications comprising of 1,686 subjects for the assessment of total cholesterol, LDL-C and HDL-C and 1,156 subjects for the assessment of triglycerides were included in the meta-analyses. Subjects who were non-carriers of a *CYP7A1* SNP (−204 A/C (rs3808607), −278 A/C (rs3808607) and rs8192875) had a greater reduction in total cholesterol (overall WMD -0.17, 95% CI -0.29, −0.06) and LDL-C levels (overall WMD -0.16, 95% CI -0.26, −0.05) as compared with subjects who borne the variant allele of *CYP7A1* SNPs when administered a statin.

**Conclusion:** The presence of variant allele of *CYP7A1* SNPs may result in suboptimal control of total cholesterol and LDL-C levels as compared with individuals who do not carry the variant allele, when administered an equivalent dose of statin.

## 1 Introduction

Good cholesterol control is vital to keep cardiovascular diseases at bay and ensure normal functioning of essential cellular processes in the body. In the human body, cholesterol is either taken up from dietary sources or predominantly synthesized *de novo* in the liver. (Lecerf and de Lorgeril, 2011). Excess cholesterol is subsequently removed via conversion into bile acids, where the process is regulated by the rate-limiting enzyme, cholesterol 7α-hydroxylase (*CYP7A1*). (Pandak et al., 2001). In conditions where cholesterol levels require drug interventions to achieve control, 3-hydroxy-3-methylglutaryl coenzyme A (HMG-CoA) reductase inhibitors, i.e., statins, are the cornerstone therapy. (Crismaru et al., 2020).

However, inter-individual differences in serum lipid responses to statin treatment have been frequently reported, of which genetics is one important intrinsic factor that contribute to these differences. There is increasing evidence to show that *CYP7A1* is highly polymorphic and patients harbouring *CYP7A1* polymorphisms, such as −204 A/C (rs3808607), −278 A/C (rs3808607) and rs8192875), experienced differential lipid lowering responses to statins. (Kajinami et al., 2005; Aruna Poduri et al., 2010; Jiang et al., 2012; Liu et al., 2017; Na Liu et al., 2020).

Multiple studies have shown that there is an association between *CYP7A1* polymorphisms and a reduced response to statin therapy, where homozygous carriers of the variant allele that encode for the reduced expression of *CYP7A1* experienced less than desirable cholesterol control when administered the same dose of statin as non-carriers. (Kajinami et al., 2005; Aruna Poduri et al., 2010; Jiang et al., 2012; Liu et al., 2017; Na Liu et al., 2020). The implication is that over time, such patients who are resistant to therapy may be subjected to a higher statin dose or polypharmacy, or experience poor cholesterol control, consequently leading to intolerable toxicities, risk of drug-drug interactions and disease progression. Because of this, there has been increasing research interest in identifying and studying functional *CYP7A1* single nucleotide polymorphisms (SNPs) and their impact across different populations.

While there is an increasing number of studies looking into this, the sample sizes of the individual studies were small, and results were inconsistent. Hence, this systematic review and meta-analysis aimed to pool the relevant studies together to provide a larger sample size for enhanced statistical power so as to ascertain any small but relevant effects of *CYP7A1* SNPs on the cholesterol lowering response to statins.

In this review, we aimed to answer the following research question: In individuals receiving statin treatment, do *CYP7A1* SNPs affect the serum lipid responses to the treatment? Specifically, we are interested in comparing the lipid response outcomes to statin treatment between carriers of the variant allele *versus* the non-variant allele of *CYP7A1* SNPs.

## 2 Methods

### 2.1 Literature search strategy

This systematic review was performed based on the Preferred Reporting Items for Systematic Reviews and Meta-analyses (PRISMA) guidelines. (Liberati et al., 2009). Medical and health sciences databases, specifically PUBMED, Cochrane and EMBASE, were used for the literature search. The literature search strategy used was adapted to suit the search framework of different databases. The search strategy for PUBMED, Cochrane and EMBASE are appended for replicability purposes (Supplementary Tables S1,S2,S3). The references of included studies were searched to identify additional relevant articles. Two independent reviewers were engaged through each phase of review and throughout the entire review process, and were responsible for the screening of study titles, reviewing of abstracts and collection of data from the full text of each report.

### 2.2 Study selection criteria

Reports of any study design that investigated the impact of genotypes of a *CYP7A1* SNP on lipid responses to a statin and published in English were included in the systematic review, without date restrictions. Studies were included if they were conducted in human participants, with neither age restrictions nor confining the indication for statin use to a specific disease. For this systematic review, we did not confine the inclusion criteria to a specific genetic model for the analysis of the association between the outcome parameters and the genotypes, but the goodness-of-fit of the genotypes to the Hardy-Weinberg equilibrium were assessed. We also did not confine the inclusion criteria to a specific intensity of statin therapy, although the intensity of statin therapy was recorded and described in accordance to American College of Cardiology (ACC) and American Heart Association (AHA) lipid guidelines. According to the ACC/AHA guidelines, statins are broadly classified into three levels: high-intensity (at least 50% reduction in LDL), moderate-intensity (30%–49% reduction in LDL) and low-intensity (less than 30% reduction in LDL) (Supplementary Table S4). (Scott et al., 2018; Donna K Arnett et al., 2019). We did not restrict the studies to a specific genotyping method for the *CYP7A1* SNPs. Pre-clinical studies, review papers and studies with insufficient data for analyses were excluded.

### 2.2 Outcomes of interest

The outcomes of interest were the treatment effects of statins on lipid parameters (namely, total cholesterol, low density lipoprotein cholesterol (LDL-C), high density lipoprotein cholesterol (HDL-C) and triglycerides (TG)) between carriers and non-carriers of *CYP7A1* SNPs.

### 2.3 Data extraction and management

A data extraction form by means of a Microsoft Excel checklist with a standardized format was used to retrieve the relevant information from the individual studies. The key data items recorded included study design, study duration, ethnicity, country, type and location of SNP, allelic and genotype status and frequencies, patient demographics, information related to treatment such as the dose, frequency and type of statins used, as well as information related to outcomes such as change from baseline lipid values or values of pre- and post-intervention lipid parameters if the former is not available.

In order to allow quantification of the primary outcome measure, the means of baseline lipid values, post administration lipid values, change from baseline lipid values, as well as standard deviations of baseline, endpoint and change from baseline, should be reported in the selected studies. If only means and standard deviations of baseline and post-administration lipid values are provided, the mean and standard deviation of the change in lipid values were calculated using the following equations:

$$Mean\;of\;the\;change\;in\;lipid\;values={Mean}_{endpoint}-{Mean}_{baseline}$$

$$Standard\;deviation\;of\;the\;change\;in\;lipid\;values=\sqrt{{SD}^{2}baseline+{SD}^{2}endpoint-\left(2\times r\times SDbaseline\times SDendpoint\right)}$$

where ‘r’ refers to the correlation coefficient that describes how the baseline and endpoint scores change in relation to each other within a group of participants. A value of 0.5 was assumed for ‘r’, and as the choice of 0.5 was arbitrary, the robustness of the study results was validated by imputing 0 and 0.8 in sensitivity analyses. (Fu, 2015).

### 2.4 Statistical analysis

The outcomes of interest were the treatment effects of statins on lipid parameters (namely, total cholesterol, low density lipoprotein cholesterol (LDL-C), high density lipoprotein cholesterol (HDL-C) and triglycerides (TG)) between carriers and non-carriers of *CYP7A1* SNPs. The treatment effects of statins ascertained through the change from baseline in lipid parameters (namely, total cholesterol, LDL-C, HDL-C and triglycerides) were meta-analysed and calculated as weighted mean differences (WMD) (with 95% CI). (Higgins JPT et al., 2022). Stratified analyses were also conducted based on ethnicity (White persons and non-White persons), SNP and the duration of statin treatment. As there is currently no consensus on a cut-off for treatment duration, the duration of statin treatment was stratified into two categories, namely, therapy of less than 12 weeks and therapy of at least 12 weeks. The Chi-square test and I^2^ statistic were employed to determine the statistical heterogeneity, where significant heterogeneity was deemed to be present if I^2^ was >40% and *p*-value was <0.10. When either condition for statistical heterogeneity was not met, the fixed-effects model was used for meta-analyses. Otherwise, a random-effects model was used to account for heterogeneity among studies. All meta-analyses were performed using Review Manager (RevMan), Version 5.4 (The Cochrane Collaboration, 2020).

## 3 Results

### 3.1 Included studies and study characteristics

**FIGURE 1:**
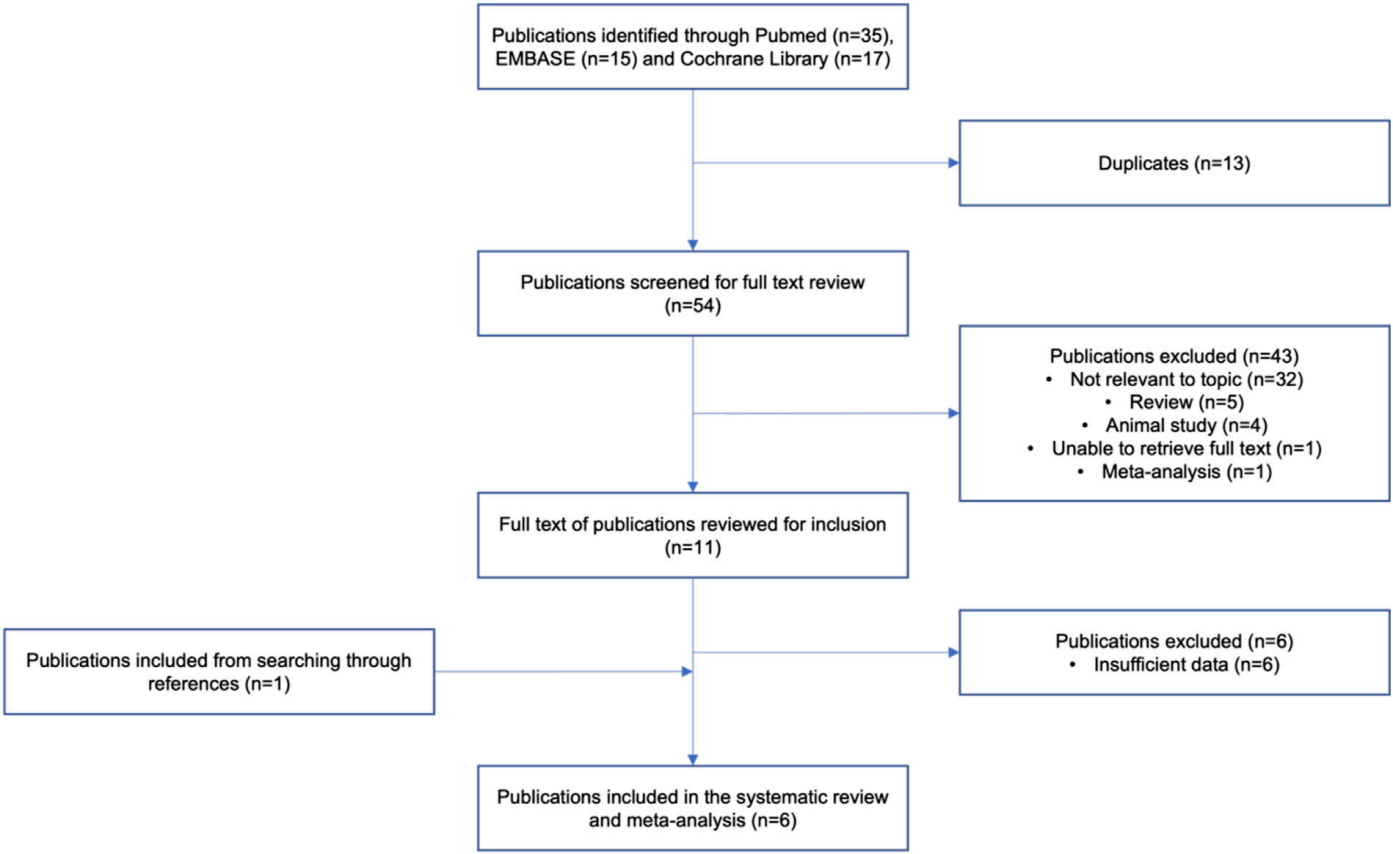
Flow chart depicting the study selection process.

A thorough electronic literature search identified 54 unique journal articles. From which, studies that were either animal studies, review papers, meta-analysis, unavailability of full text or deemed irrelevant to the topic were excluded (Figure 1). A total of 11 studies were included after screening of titles and abstracts. Upon a comprehensive review of the article contents, a total of 6 studies were further excluded due to insufficient data for meta-analysis. One additional study was identified to be relevant to the topic in the process of the detailed review, which resulted in a total of 6 articles being included in the systematic review and analysis. The included studies were published between 2005 and 2021 (Table 1) (Hofman MK et al., 2005; Kajinami et al., 2005; Aruna Poduri et al., 2010; Wei et al., 2011; Liu et al., 2017; Abed et al., 2021). Two of these studies were cohort studies, 2 were secondary analyses of randomized controlled trials and 2 were cross-sectional studies (Hofman MK et al., 2005; Kajinami et al., 2005; Aruna Poduri et al., 2010; Wei et al., 2011; Liu et al., 2017; Abed et al., 2021). A total of 1,686 subjects were included for the assessment of total cholesterol, LDL-C and HDL-C, while a total of 1,156 subjects were included for the assessment of triglycerides in the analysis. Five studies ((Kajinami et al., 2005; Aruna Poduri et al., 2010; Wei et al., 2011; Liu et al., 2017; Abed et al., 2021)) were conducted with atorvastatin and 1 study ((Hofman MK et al., 2005)) was conducted with pravastatin, with a moderate (LDL-C lowering of 30%–49%) to high (LDL-C lowering of ≥50%) dose intensity administered in all studies. In terms of study duration, 3 studies ((Hofman MK et al., 2005; Kajinami et al., 2005; Abed et al., 2021)) had a treatment duration of at least 12 weeks and 3 studies ((Aruna Poduri et al., 2010; Wei et al., 2011; Liu et al., 2017)) had a treatment duration of less than 12 weeks. Across the different SNPs analysed across the meta-analysis, 538 subjects carried the −204 A>C polymorphism, 342 subjects carried the −278 A>C polymorphism and 9 subjects carried the rs8192875 polymorphism. We noted that genotyping of *CYP7A1* SNPs in the studies was mostly carried by polymerase chain reaction (PCR) - based methods such as the PCR - restriction fragment length polymorphism (PCR-RFLP) method, with further validation by DNA sequencing (Table 1). (Hofman MK et al., 2005; Kajinami et al., 2005; Aruna Poduri et al., 2010; Wei et al., 2011; Liu et al., 2017; Abed et al., 2021).

**TABLE 1:** Characteristics of included studies (n = 6).

References, year	Study design	No. of subjects (AA/AB + BB)^a^	Mean age (aa/AB/BB)^a^	Gender (AA/AB + BB)^a^	Single nucleotide polymorphism studied	Genotyping method	Drug used	Duration of treatment	Ethnicity	Background disease condition		Outcome measures
Kajinami et al., 2005 (Kajinami et al., 2005)	Secondary analyses of a randomized controlled trial	96/228	Male: 56 ± 12/57 ± 11/53 ± 11; Female: 61 ± 12/61 ± 10/58 ± 11	Male: 59/136 Female: 37/92	−204 A>C (rs3808607)	Polymerase chain reaction—restriction fragment length polymorphism (PCR-RFLP)	Atorvastatin 10 mg once daily	52 weeks	Mostly Caucasians (92%)		Primary hypercholesterolemia	Total cholesterol, LDL-C^b^, HDL-C^c^, triglycerides
Hofman et al., 2005 (Hofman MK et al., 2005)	Secondary analyses of a randomized controlled trial	155/208	57 ± 8/56 ± 8/56 ± 8	All males	−278 A>C (rs3808607)	PCR-RFLP and PCR followed by use of sequence detection system	Pravastatin 40 mg once daily	96 weeks	Caucasians		Coronary Artery Disease	Total cholesterol, LDL-C^b^, HDL-C^c^, triglycerides
Poduri et al., 2010 (Aruna Poduri et al., 2010)	Cross-sectional study	−204 A>C: 116/149–278 A>C: 131/134	47.52 ± 7.7^d^	Male: 222^d^ Female: 43^d^	−204 A>C, −278 A>C (rs3808607)	PCR-RFLP, further validated by random DNA sequencing	Atorvastatin 20 mg once daily	6 weeks	Indian		Coronary Artery Disease	Total cholesterol, LDL-C^b^, HDL-C^c^
Wei et al., 2011 (Wei et al., 2011)	Cohort study	55/126	53 ± 8.04/54 ± 7.77/57 ± 8.30	All males	−204 A>C (rs3808607)	PCR-RFLP	Atorvastatin 20 mg once daily	4 weeks	Chinese		Hyperlipidaemia	Total cholesterol, LDL-C^b^, HDL-C^c^, triglycerides
Liu et al., 2017 (Liu et al., 2017)	Cohort study	162/9	59.46 ± 8.21/57.22 ± 2.19/Nil	Male: 118/5 Female: 42/4	rs8192875	iPLEX assay in conjunction with the MassARRAY platform	Routine—Less than atorvastatin 20 mg once daily Intensive - atorvastatin 80 mg once daily for 2 days before and 40 mg/day for 28 days after procedure	30 days	Chinese		Non-ST Elevation Myocardial Infarction (NSTEMI)/Stable angina pectoris	Total cholesterol, LDL-C^b^, HDL-C^c^, triglycerides
Abed et al., 2021 (Abed et al., 2021)	Cross-sectional study	82/35	54.04 ± 9.71^d^	Male: 69^d^ Female: 48^d^	−204 A>C (rs3808607)	PCR-RFLP	Atorvastatin 20 mg once daily	12 weeks	Jordanian		Type 2 Diabetes Mellitus	Total cholesterol, LDL-C^b^, HDL-C^c^, triglycerides

^a^ AA, refers to the non-carriers of a CYP7A1 SNP, while AB, and BB, refers to carriers of a CYP7A1 SNP.

^b^ LDL-C, low-density lipoprotein cholesterol.

^c^ HDL-C, high-density lipoprotein cholesterol.

^d^ Demographics were not stratified by genotype; only overall information was present.

### 3.2 Outcomes of interest

#### 3.2.1 Treatment effect of statins on total cholesterol

**FIGURE 2:**
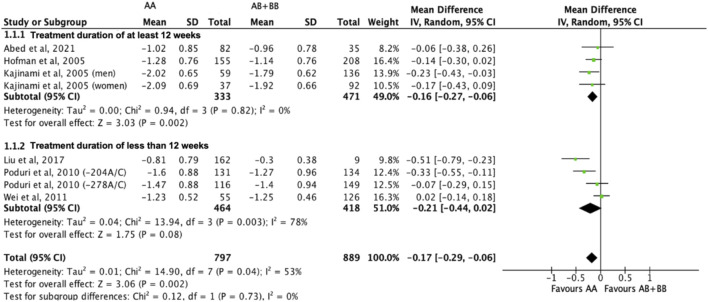
Mean difference in total cholesterol lowering response to statins between carriers (AB + BB) *versus* non-carriers (AA) of CYP7A1 SNPs using a random-effects model, stratified by treatment duration.

**FIGURE 3:**
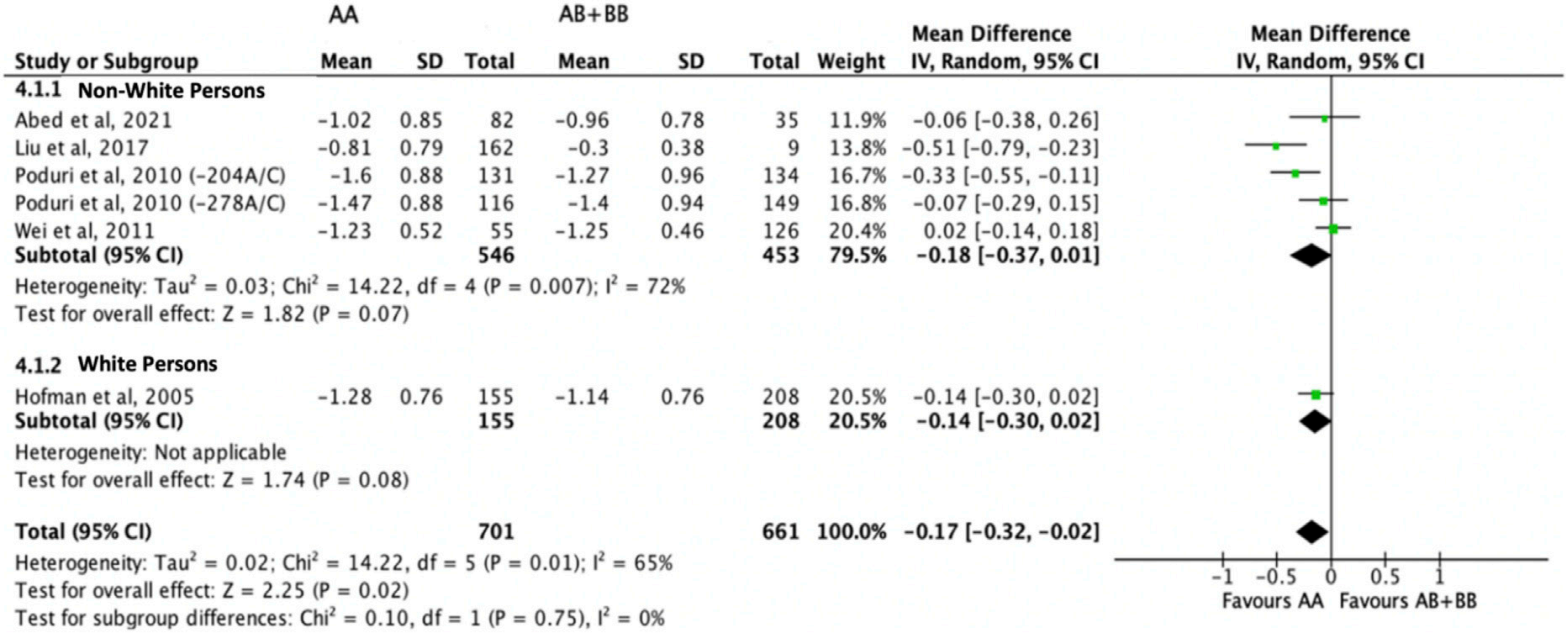
Mean difference in total cholesterol lowering response to statins between carriers (AB + BB) *versus* non-carriers (AA) of CYP7A1 SNPs using a random-effects model, stratified by ethnicity.

**FIGURE 4:**
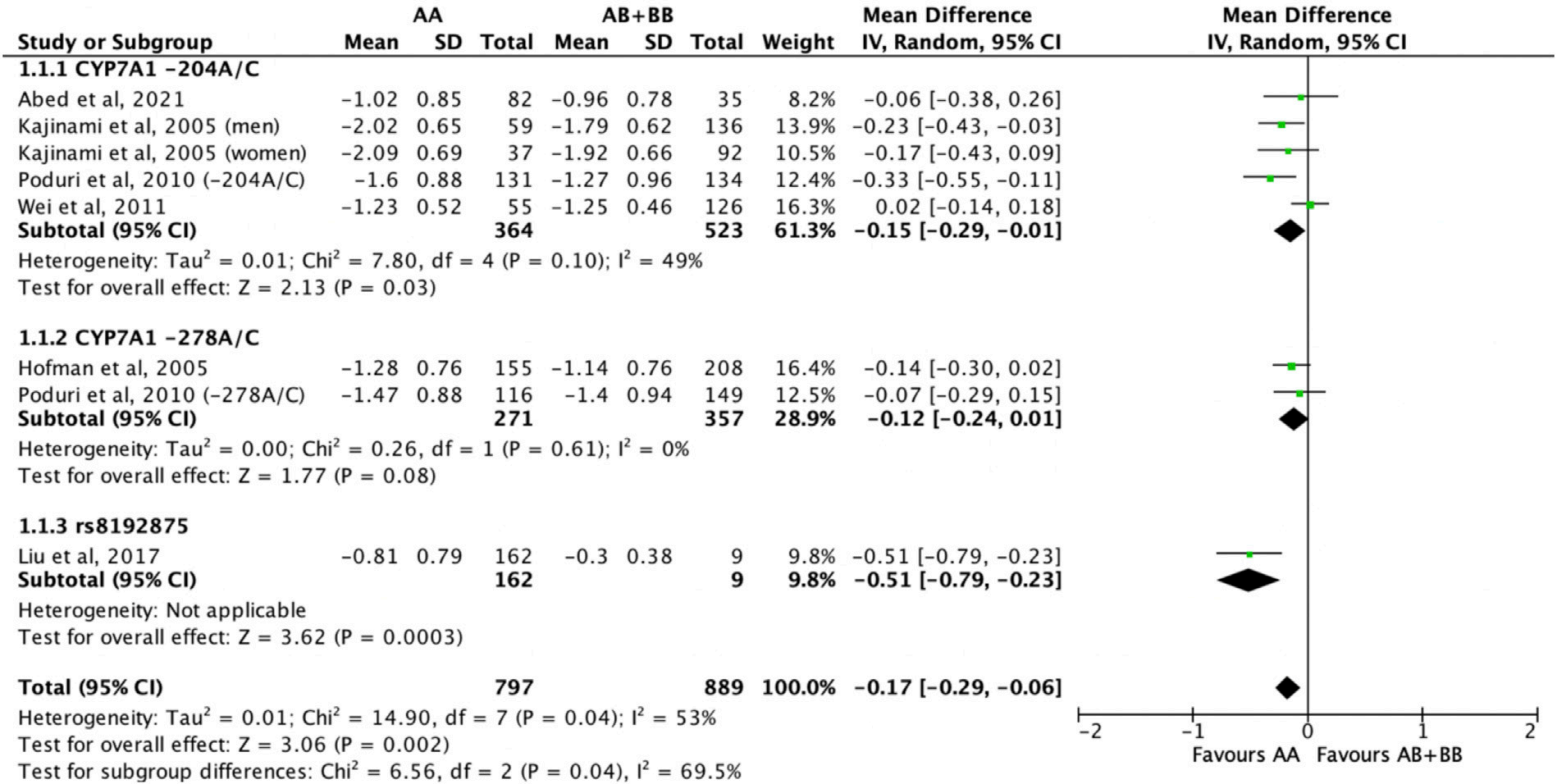
Mean difference in total cholesterol lowering response to statins between carriers (AB + BB) *versus* non-carriers (AA) of CYP7A1 SNPs using a random-effects model, stratified by CYP7A1 SNP.

Subjects who were non-carriers of a *CYP7A1* SNP (A allele of rs3808607 and G allele of rs8192875) had a significantly greater reduction in total cholesterol levels as compared with subjects who borne the variant allele of *CYP7A1* SNPs (C allele of rs3808607 and A allele of rs8192875) when administered a statin (overall WMD -0.17, 95% CI -0.29, −0.06) (Figure 2). Subjects who were non-carriers of a *CYP7A1* SNP (A allele of rs3808607 and G allele of rs8192875) and had received at least 12 weeks of statin therapy had a significantly greater reduction in total cholesterol levels as compared with subjects who borne the variant allele of *CYP7A1* SNPs (C allele of rs3808607 and A allele of rs8192875) (WMD -0.16, 95% CI -0.27, −0.06) (Figure 2). However, there was no significant difference in reduction of total cholesterol levels among those receiving less than 12 weeks of therapy (WMD -0.21, 95% CI -0.44, 0.02) (Figure 2). Similarly, the ethnicity of the subjects did not have a significant impact on the reduction of total cholesterol levels among the Non-White persons (WMD -0.18, 95% CI -0.37, 0.01) and White persons (WMD -0.14, 95% CI -0.30, 0.02), respectively (Figure 3). When stratified by *CYP7A1* SNP, statistically significant reductions in total cholesterol levels of subjects who were non-carriers of a *CYP7A1* SNP were observed among those with *CYP7A1* -204A/C (WMD -0.15, 95% CI -0.29, −0.01) and rs8192875 (WMD -0.51, 95% CI -0.79, −0.23) but not among those with *CYP7A1* -278A/C (WMD -0.12, 95% CI -0.24, 0.01) (Figure 4).

#### 3.2.2 Treatment effect of statins on LDL-C

**FIGURE 5:**
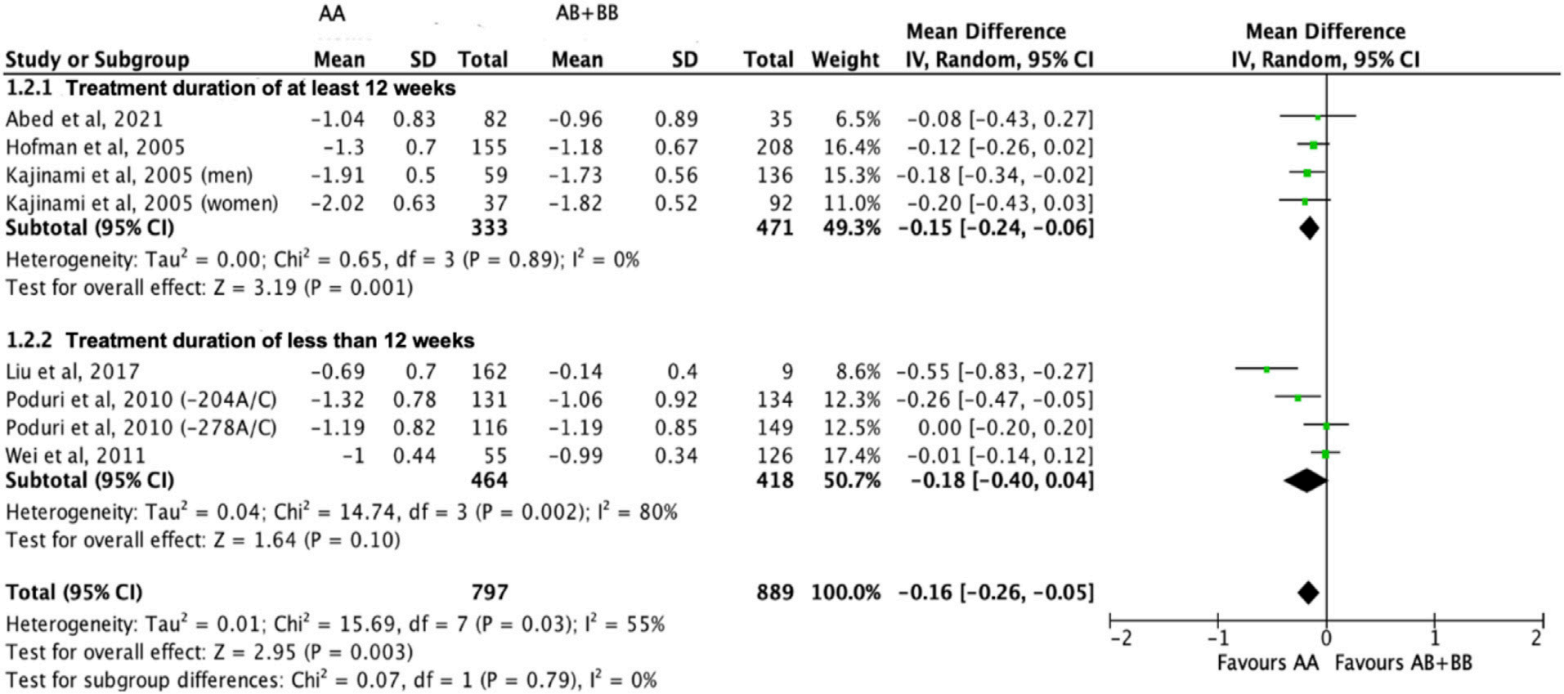
Mean difference in LDL-C lowering response to statins between carriers (AB + BB) *versus* non-carriers (AA) of CYP7A1 SNPs using a random-effects model, stratified by treatment duration.

**FIGURE 6:**
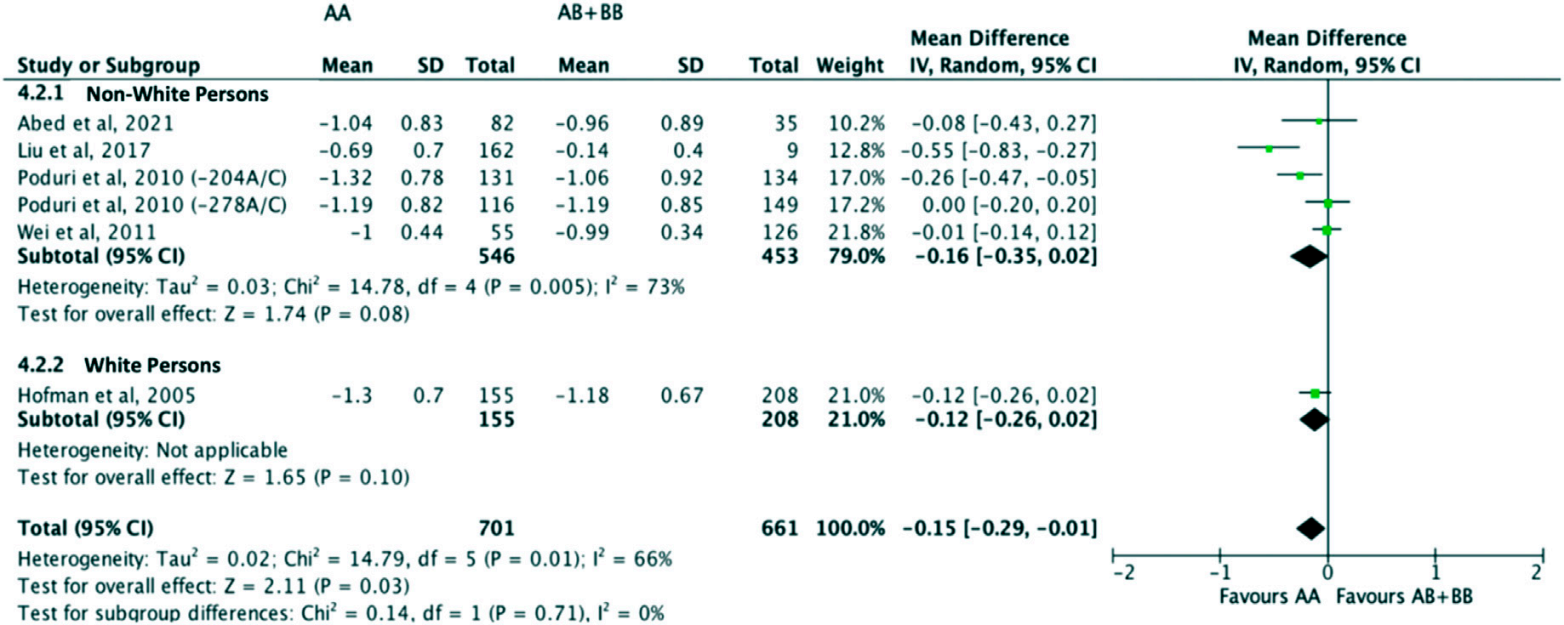
Mean difference in LDL-C lowering response to statins between carriers (AB + BB) *versus* non-carriers (AA) of CYP7A1 SNPs using a random-effects model, stratified by ethnicity.

**FIGURE 7:**
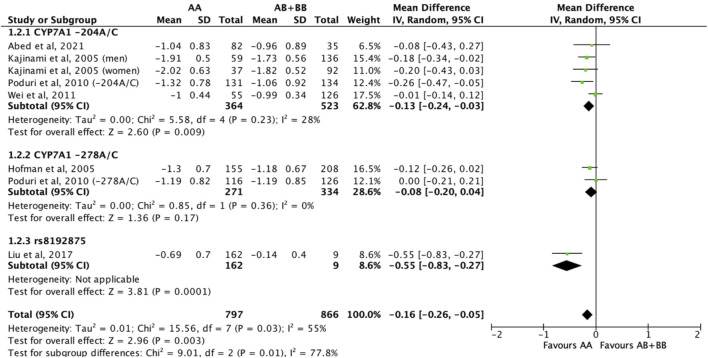
Mean difference in LDL-C lowering response to statins between carriers (AB + BB) *versus* non-carriers (AA) of CYP7A1 SNPs using a random-effects model, stratified by CYP7A1 SNP.

Subjects who were non-carriers of a *CYP7A1* SNP (A allele of rs3808607 and G allele of rs8192875) had a significantly greater reduction in LDL-C levels as compared with subjects who borne the variant allele of *CYP7A1* SNPs (C allele of rs3808607 and A allele of rs8192875) when administered a statin (overall WMD -0.16, 95% CI -0.26, −0.05) (Figure 5). Similar to the analyses on total cholesterol, subjects who were non-carriers of a *CYP7A1* SNP (A allele of rs3808607 and G allele of rs8192875) and had received at least 12 weeks of statin therapy had a significantly greater reduction in LDL-C levels as compared to subjects who borne the variant allele of *CYP7A1* SNPs (C allele of rs3808607 and A allele of rs8192875) (WMD -0.15, 95% CI -0.24, −0.06) (Figure 5). However, there was no significant difference in reduction of total cholesterol levels among those receiving less than 12 weeks of therapy (WMD -0.18, 95% CI -0.40, 0.04) (Figure 5). When stratified by ethnicity, there was no significant difference in reduction of LDL-C levels among Non-White persons (WMD -0.16, 95% CI -0.35, 0.02) and White persons (WMD -0.12, 95% CI -0.26, 0.02), respectively (Figure 6). When stratified by *CYP7A1* SNP, statistically significant reductions in LDL-C levels of subjects who were non-carriers of a *CYP7A1* SNP were observed among those with *CYP7A1* -204A/C (WMD -0.13, 95% CI -0.24, −0.03) and rs8192875 (WMD -0.55, 95% CI -0.83, −0.27) but not among those with CYP7A1 -278A/C (WMD -0.08, 95% CI -0.20, 0.04) (Figure 7).

#### 3.2.3 Treatment effect of statins on HDL-C

**FIGURE 8:**
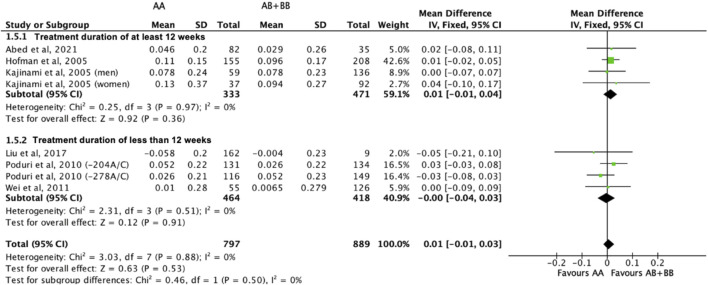
Mean difference in HDL-C improvement response to statins between carriers (AB + BB) *versus* non-carriers (AA) of CYP7A1 SNPs using a fixed-effects model, stratified by treatment duration.

**FIGURE 9:**
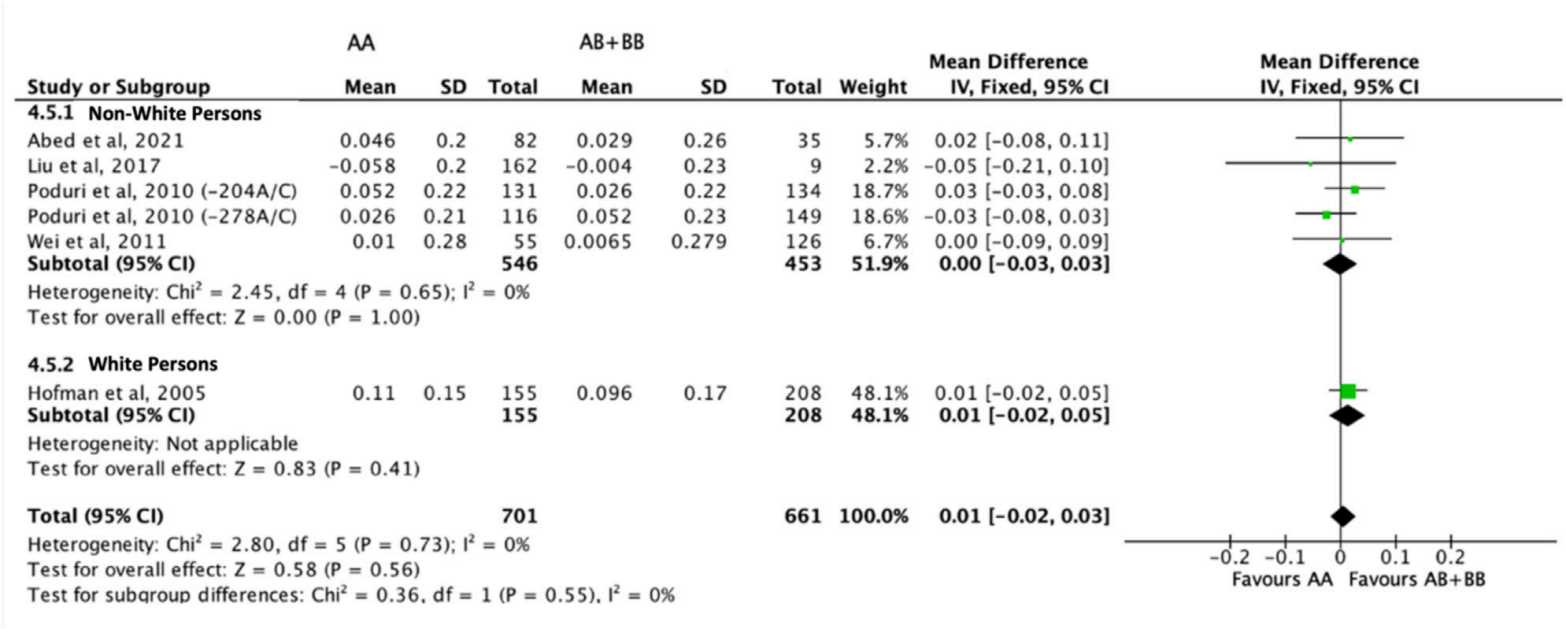
Mean difference in HDL-C improvement response to statins between carriers (AB + BB) *versus* non-carriers (AA) of CYP7A1 SNPs using a fixed-effects model, stratified by ethnicity.

**FIGURE 10:**
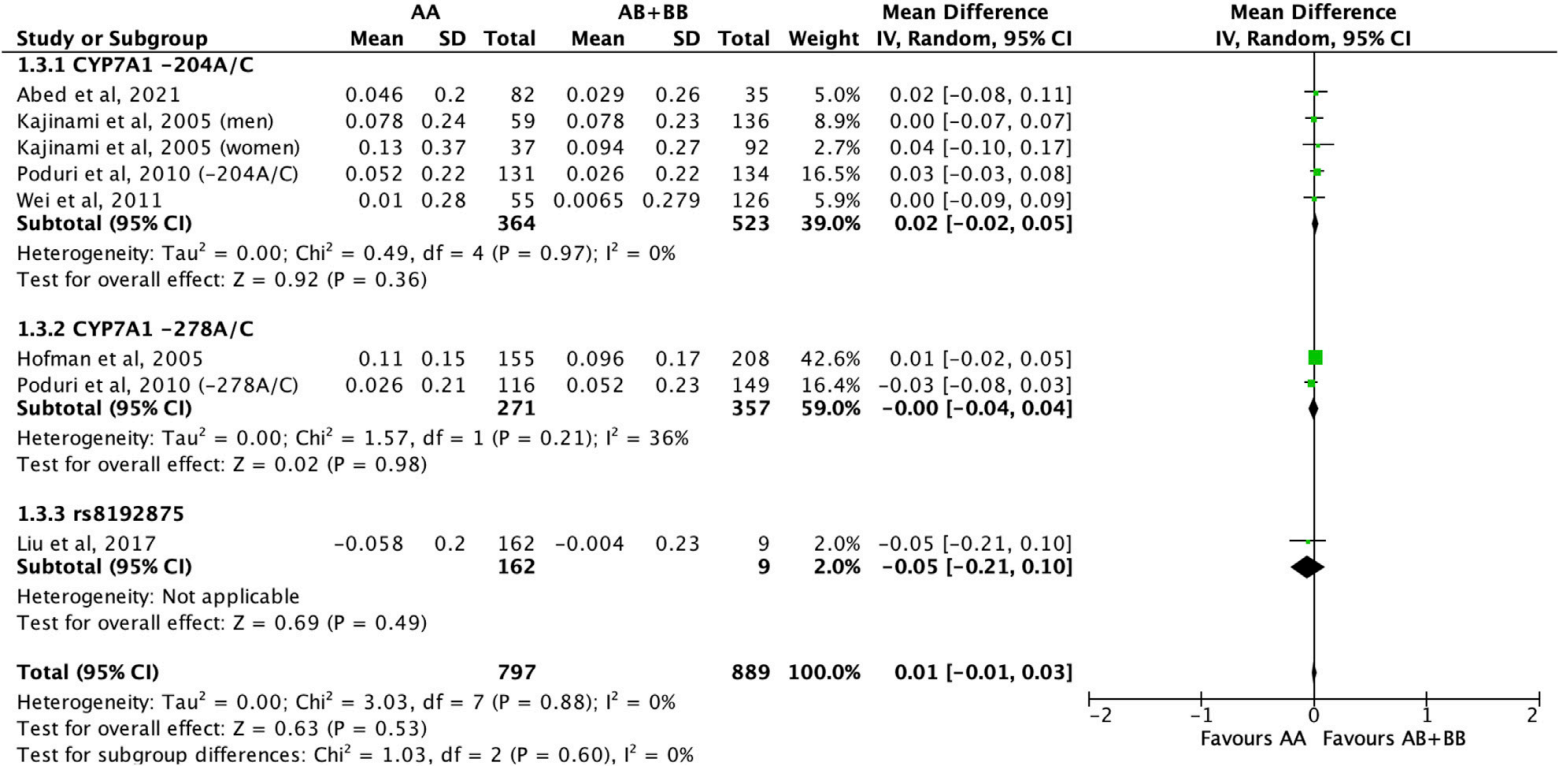
Mean difference in HDL-C improvement response to statins between carriers (AB + BB) *versus* non-carriers (AA) of CYP7A1 SNPs using a fixed-effects model, stratified by CYP7A1 SNP.

There was no differential impact of statins on HDL-C levels between subjects who were non-carriers of a *CYP7A1* SNP (A allele of rs3808607 and G allele of rs8192875) and subjects who borne the variant allele of *CYP7A1* SNPs (C allele of rs3808607 and A allele of rs8192875) (WMD 0.01, 95% CI -0.01, 0.03) overall (Figure 8). Similar results were observed when stratified by duration of therapy (less than 12 weeks of therapy: WMD -0.00, 95% CI -0.04, 0.03; at least 12 weeks of therapy: WMD 0.01, 95% CI -0.01, 0.04) (Figure 8), by ethnicity of the subjects (Non-White persons: WMD 0.00, 95% CI -0.03, 0.03; White persons: WMD 0.01, 95% CI -0.02, 0.05) (Figure 9) or by SNP (*CYP7A1* -204A/C: WMD 0.02, 95% CI -0.02, 0.05; *CYP7A1* -278A/C: WMD -0.00, 95% CI -0.04, 0.04; rs8192875: WMD -0.05, 95% CI -0.21, 0.10) (Figure 10).

#### 3.2.4 Treatment effect of statins on triglycerides

**FIGURE 11:**
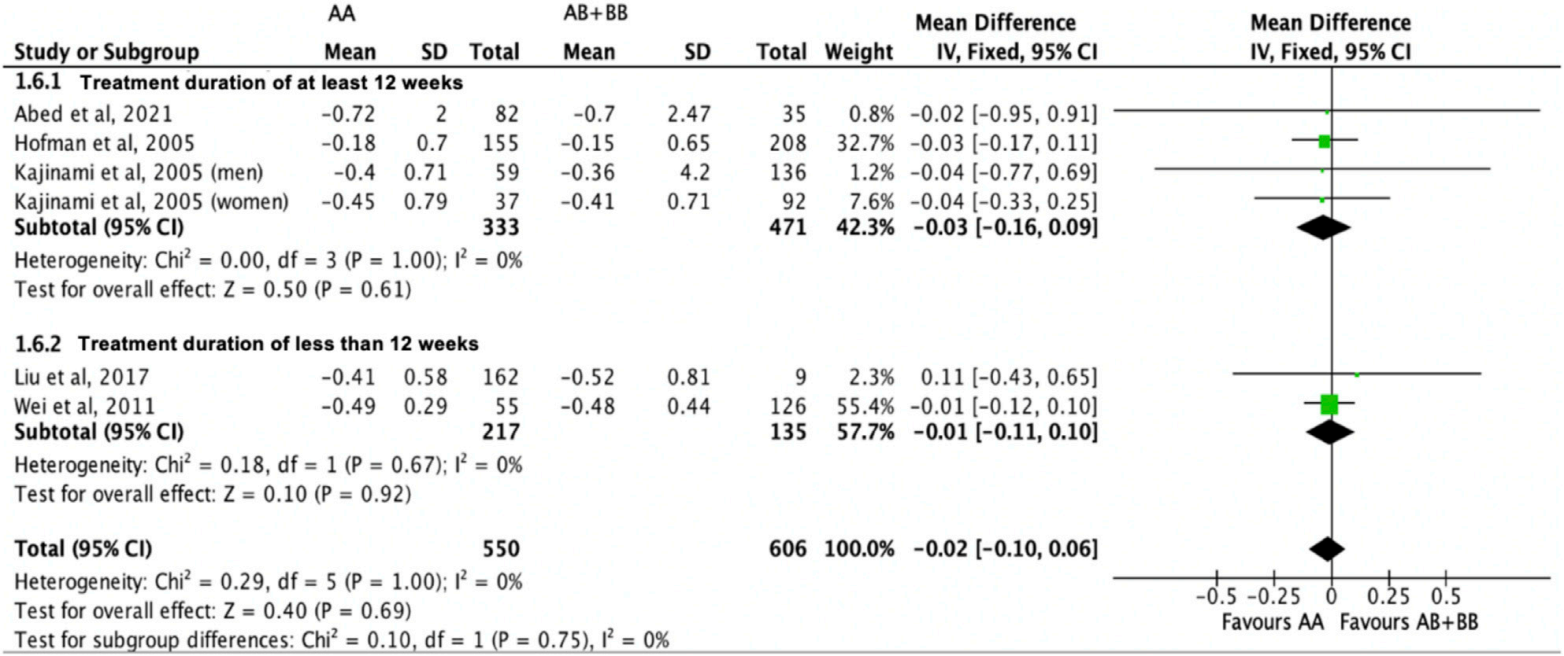
Mean difference in triglycerides lowering response to statins between carriers (AB + BB) *versus* non-carriers (AA) of CYP7A1 SNPs using a fixed-effects model, stratified by treatment duration.

**FIGURE 12:**
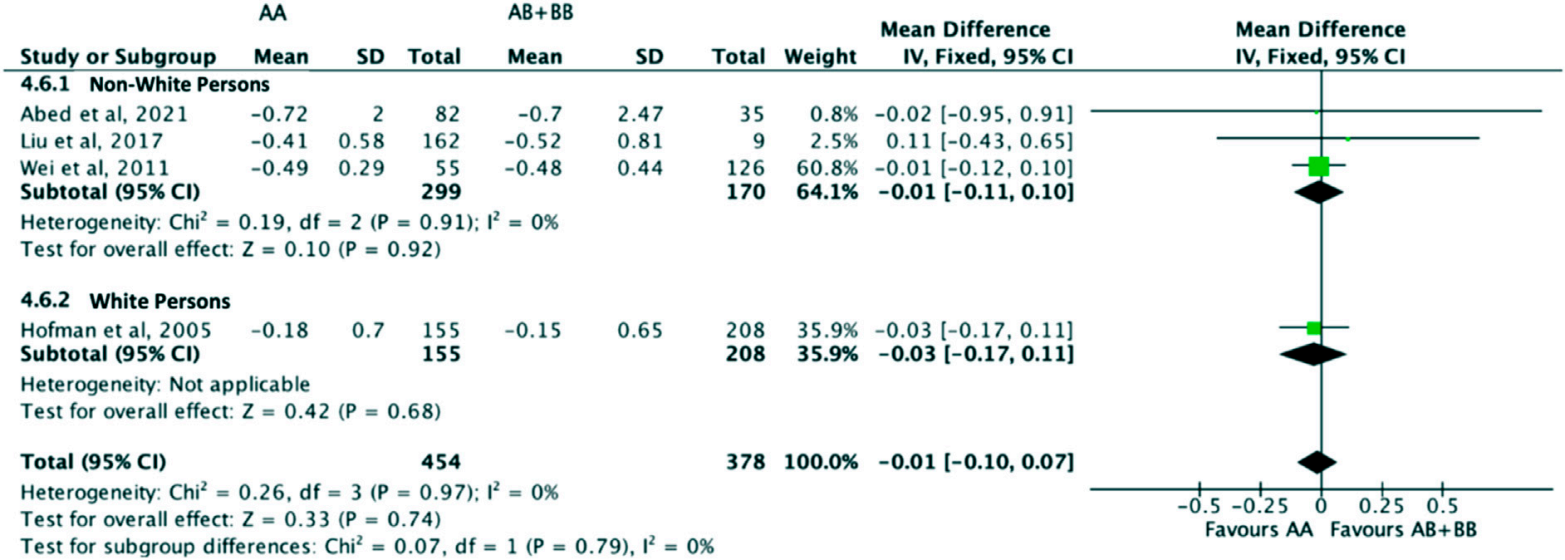
Mean difference in triglycerides lowering response to statins between carriers (AB + BB) *versus* non-carriers (AA) of CYP7A1 SNPs using a fixed-effects model, stratified by ethnicity.

**FIGURE 13:**
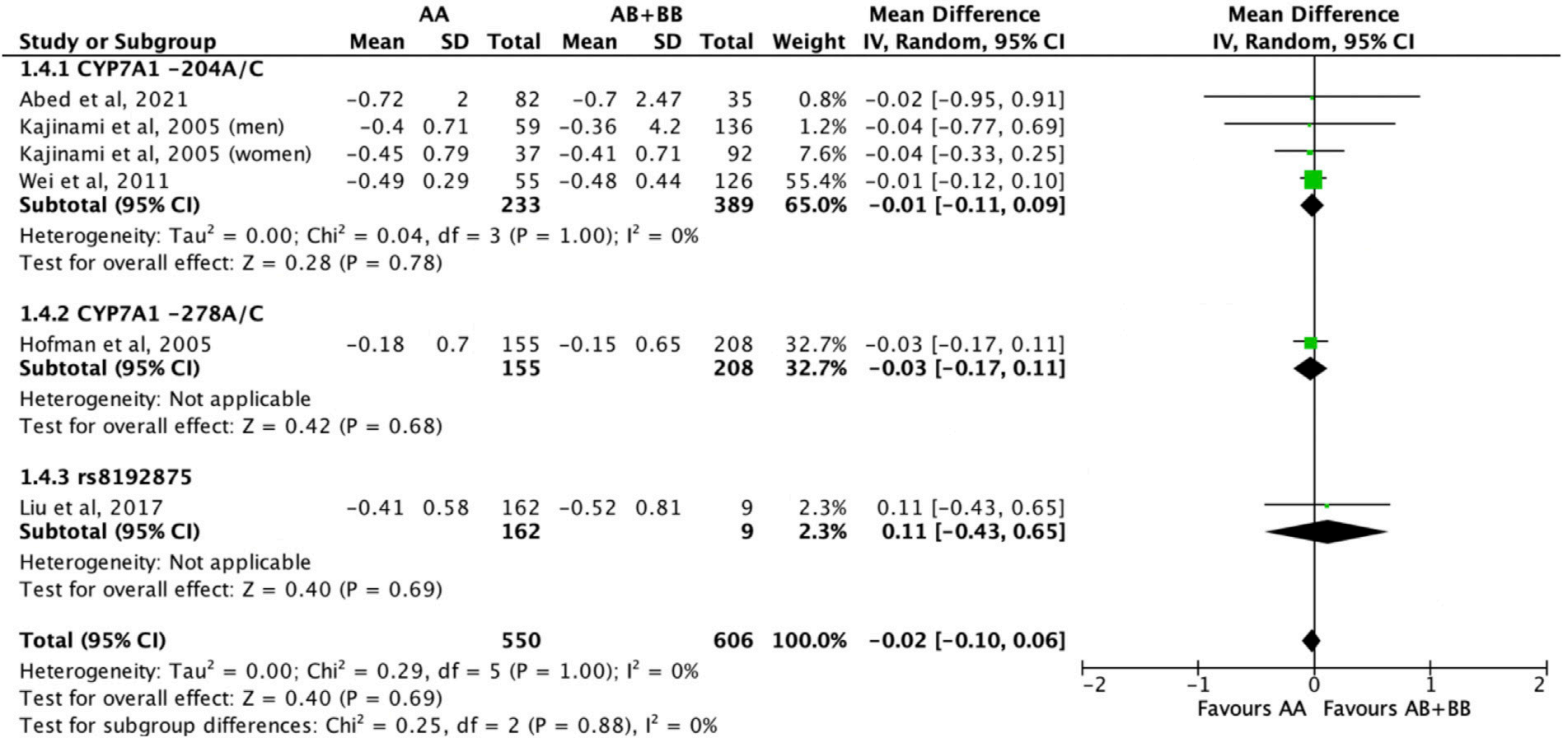
Mean difference in triglycerides lowering response to statins between carriers (AB + BB) *versus* non-carriers (AA) of CYP7A1 SNPs using a fixed-effects model, stratified by CYP7A1 SNP.

Similarly, the treatment effect of statins on triglyceride levels was not significantly different between subjects who were non-carriers of a *CYP7A1* SNP (A allele of rs3808607 and G allele of rs8192875) and subjects who borne the variant allele of *CYP7A1* SNPs (C allele of rs3808607 and A allele of rs8192875) (WMD -0.02, 95% CI -0.10, 0.06) overall (Figure 11). There was also no differential impact of statins on triglyceride levels between carriers and non-carriers of *CYP7A1* SNPs when stratified by duration of therapy (less than 12 weeks of therapy: WMD -0.01, 95% CI -0.11, 0.10; at least 12 weeks of therapy: WMD -0.03, 95% CI -0.16, 0.09) (Figure 11), by ethnicity (Non-White persons: WMD -0.01, 95% CI -0.11, 0.10; White persons: WMD -0.03, 95% CI -0.17, 0.11) (Figure 12) or by SNP (*CYP7A1* -204A/C: WMD -0.01, 95% CI -0.11, 0.09; *CYP7A1* -278A/C: WMD -0.03, 95% CI -0.17, 0.11; rs8192875: WMD 0.11, 95% CI -0.43, 0.65) (Figure 13).

#### 3.2.5 Sensitivity analyses

When the correlation coefficient was arbitrarily imputed with ‘0′and ‘0.8′, results remained consistent with the overall trends observed in the primary analyses when a correlation coefficient of ‘0.5′was used, demonstrating robustness in the results obtained (Supplementary Figures S1-S8).

## 4 Discussion

This meta-analysis serves as an update to a previously published review that meta-analysed the results from 3 studies to evaluate the impact of one specific *CYP7A1* SNP, i.e., −204 A/C, on the treatment effects of statins on total cholesterol, LDL-C, HDL-C and triglycerides (Hofman MK et al., 2005; Kajinami et al., 2005; Aruna Poduri et al., 2010; Wei et al., 2011; Li et al., 2014). It is an important verification of earlier trends by analysing a more recent and larger data set. To the best of our knowledge, this is the first meta-analysis that pooled relevant studies without restricting to a specific *CYP7A1* SNP. This not only allows us to amass a larger sample size for better statistical power and more precise point estimates, but also provides us with insights on how these *CYP7A1* SNPs can collectively affect lipid outcomes in subjects with or without the variant allele who were administered a statin. Additionally, we expanded our scope of review to look into the effect of treatment duration on the lipid parameters through a stratified analysis, which was not explored in an existing review (Li et al., 2014).

A total of 6 studies comprising 1,686 subjects for the assessment of total cholesterol, LDL-C and HDL-C, and 1,156 subjects for the assessment of triglycerides were included in this meta-analysis. The study findings showed that subjects who were non-carriers of a *CYP7A1* SNP had a statistically significantly greater reduction in total cholesterol and LDL-C levels as compared to their counterparts who borne the variant allele of *CYP7A1* SNPs when administered a statin, irrespective of the duration of therapy. This implied that patients who carry the variant allele of *CYP7A1* SNPs are at risk of a reduced treatment efficacy when administered an equivalent dose of statin as compared to patients who do not carry the variant allele. Consequentially, these patients might require a higher than necessary statin dose to achieve a similar reduction in total cholesterol and LDL-C levels, and be predisposed to the detrimental side effects of statins such as rhabdomyolysis. (Noyes et al., 2021). One possible explanation for the observed differences in reduction of total cholesterol and LDL-C levels could be that there is a reduced expression of *CYP7A1* in subjects who borne a variant allele of a *CYP7A1* SNP, which in turn reduces the metabolism of cholesterol to bile acids in the liver thereby leading to accumulation of cholesterol. (Wang et al., 2018; Chiang and Ferrell, 2020). A reduced expression of *CYP7A1* may also downregulate LDL-receptor activity leading to reduced catabolism of LDL-C. (Pullinger et al., 2002). Our study results are also congruent with the previously published meta-analysis where subjects who were non-carriers of a *CYP7A1* SNP were shown to have a greater reduction in total cholesterol (MD -2.56, 95% CI -4.49, −0.63, *p* = 0.009; n = 3) and LDL-C levels (MD -0.33, 95% CI -0.26, 1.41, *p* = 0.71; n = 3) as compared to their counterparts who borne a variant allele of *CYP7A1* -204 A>C (Li et al., 2014). While the previously published meta-analysis did not establish a significant difference in LDL-C levels between carriers and non-carriers of *CYP7A1* -204A/C when administered a statin, our study reported a significant difference in reduction of LDL-C levels in favour of non-carriers of *CYP7A1* -204A/C.

There is, however, insufficient evidence to suggest that the presence of variant allele of *CYP7A1* SNPs had a significant effect on the reduction of triglycerides levels or improvement in HDL-C levels when subjects were administered a statin. This is in contrast to the previously published meta-analysis where subjects who were non-carriers of a *CYP7A1* SNP had a greater reduction in triglycerides (MD -1.92, 95% CI -7.48, 3.63, *p* = 0.64; n = 2) and a greater increase in HDL-C levels (MD -0.39, 95% CI -1.13, 0.35, *p* = 0.30; n = 3) as compared to their counterparts who borne a variant allele of a *CYP7A1* SNP (Li et al., 2014). One plausible explanation for the lack of a significant effect on HDL-C could be that statins generally have a mild effect on HDL-C, which resulted in minimal change from baseline across all subjects and consequentially, negligible differences between subjects with the homozygous dominant genotype and variant allele genotype. This is not surprising as statins are known to have a modest effect on HDL-C and other pharmacologic agents (e.g., niacin and fibrates) in place of statins are routinely used in practice to improve HDL-C levels (Chapman, 2004; Schachter, 2004; RobertOh, 2007; Amit and Khera, 2013; Parhofer KG and Laufs, 2019). Further, *CYP7A1* is not a key enzyme involved in the regulation of triglycerides levels, and therefore perturbation of its bioactivity via genetic polymorphism may not exhibit direct impact on triglycerides metabolism.

We were also able to learn from the study results that a longer treatment duration with statins (at least 12 weeks), at this juncture, has a favourable impact on total cholesterol and LDL-C levels in subjects who were non-carriers of a *CYP7A1* SNP as compared to subjects with the variant allele of the *CYP7A1* SNPs. This also reflects the clinical significance of *CYP7A1* genotype in the long term management of cholesterol, which is a more plausible scenario than as an acute treatment. However, duration of therapy did not have a significant impact on HDL-C and triglycerides levels. Future studies, if available, can be conducted to further elucidate the association between study duration and treatment effect of statins on all four lipid parameters in subjects with or without the variant allele of the *CYP7A1* SNPs.

We highlighted earlier that a previously published meta-analysis focused on one specific *CYP7A1* SNP, i.e., −204 A/C, while our meta-analysis was conducted on all relevant studies without restricting to a specific *CYP7A1* SNP. Specifically, the studies included in our meta-analysis looked into the *CYP7A1* SNPs rs3808607 and rs8192875. The *CYP7A1* SNP, rs3808607, is located in the promoter regions, while rs8192875 is located on the fourth exon of the gene and is associated with an amino acid change from aspartic acid to asparagine. It is known that the promoter region of the *CYP7A1* gene contains multiple functional binding sites for liver-enriched transcription factors. (Molowa DT et al., 1992; Cooper et al., 1997; Deng et al., 2017). It is therefore possible that a polymorphism in this region can inhibit the binding of transcription factors thereby decreasing transcription and resulting in a reduced expression of the *CYP7A1* enzyme. It is also suggested in literature that *CYP7A1* expression can possibly be modulated by two interacting regulatory SNPs, specifically an enhancer SNP and promoter SNP, adding weight to our perspective that it is meaningful to examine the pooled impact of *CYP7A1* SNPs on lipid outcomes. (Wang et al., 2018). As for exonal SNPs, they have the ability to replace the amino acid that encodes a protein and potentially alter the amino acid sequence such that protein structures and functions are impacted. (Deng et al., 2017). Therefore, by including studies without restricting to a specific *CYP7A1* SNP to the meta-analysis, we not only can increase the sample size for a meaningful statistical analysis but can also better appreciate how these *CYP7A1* SNPs can collectively affect the lipid outcomes in subjects with or without the variant allele of the *CYP7A1* SNPs who were administered a statin.

Nonetheless, our study has the following potential limitations that should be taken into consideration when interpreting the study results. Firstly, we were not able to assess the impact of statin intensity on the lipid outcomes between the two groups of subjects as a majority of the included studies used a moderate-intensity statin. Future studies can be conducted using either low or high doses of statins to allow a more holistic assessment of the impact of a differential statin dose. Secondly, we were also not able to assess the impact of different statins on the lipid outcomes. A total of 5 out of 6 studies ((Kajinami et al., 2005; Aruna Poduri et al., 2010; Wei et al., 2011; Liu et al., 2017; Abed et al., 2021) used atorvastatin, while the remaining study ((Hofman MK et al., 2005)) used pravastatin. Considering different statins have different chemical structures which in turn affect their pharmacokinetic-pharmacodynamic properties and potentially efficacy and safety profiles, conducting future studies with a diverse range of statins will aid in providing more comprehensive and meaningful results (Schachter, 2004). Future research should be conducted to shed light on the impact of different statins and statin doses on the lipid outcomes in subjects with or without variant allele of *CYP7A1* SNPs. Thirdly, many non-genetic extrinsic factors, such as comorbidities and comedications, can interact with the genotypes resulting in a phenomenon known as phenoconversion. (Shah and Smith, 2015). Phenoconversion converts genotypic extensive metabolisers (EMs) into phenotypic poor metabolizers (PMs) of drugs, thereby modifying patients’ clinical response and can significantly impact the interpretation of genotype-focused studies. (Shah and Smith, 2015). It will be prudent to consider patients’ clinical features and medical history when considering the impact of *CYP7A1* SNPs on their lipid outcomes with the administration of statins.

## 5 Conclusion

Our meta-analysis showed that the presence of variant alleles of CYP7A1 SNPs can result in a smaller reduction in total cholesterol and LDL-C levels as compared with individuals who do not carry the variant allele, when administered an equivalent dose of statin. There was, however, no significant impact of variant alleles of *CYP7A1* SNPs on triglycerides and HDL-C levels when subjects were administered an equivalent dose of statin. Taken together, the exact pharmacogenetic relevance of CYP7A1 SNPs to statin therapy awaits wider clinical association and mechanistic investigation.

## Data Availability

The original contributions presented in the study are included in the article/Supplementary Material, further inquiries can be directed to the corresponding author.

## References

[B1] Abed E. J. Y. Alhawari H. Abdullah S. Zihlif M. (2021). How the cytochrome 7a1 (CYP7A1) and ATP-binding cassette G8 (ABCG8) genetic variants affect atorvastatin response among type 2 diabetic patients attending the University of Jordan Hospital. Int. J. Clin. Pharmacol. Ther. 59 (2), 99–108. 10.5414/CP203779 33074092

[B2] Amit V. Khera J. P. (2013). Management of low levels of high-density lipoprotein-cholesterol. Circulation 128 (1), 72–78. 10.1161/circulationaha.112.000443 23817482 PMC4231714

[B3] Aruna Poduri M. K. Ajay B. Sehrawat B. S. Sharma Yashpaul Talwar Kewal K. (2010). Common variants of HMGCR, CETP, APOAI, ABCB1, CYP3A4, and CYP7A1 genes as predictors of lipid-lowering response to atorvastatin therapy. DNA Cell. Biol. 29, 629–637. 10.1089/dna.2009.1008 20578904

[B4] Chapman M. J. (2004). Are the effects of statins on HDL-cholesterol clinically relevant? Eur. Heart J. Suppl. 6, C58–C63. 10.1016/j.ehjsup.2004.04.002

[B5] Chiang J. Y. L. Ferrell J. M. (2020). Up to date on cholesterol 7 alpha-hydroxylase (CYP7A1) in bile acid synthesis. Liver Res. 4 (2), 47–63. 10.1016/j.livres.2020.05.001 34290896 PMC8291349

[B6] Cooper A. D. C. J. Botelho-Yetkinler M. J. Cao Y. Taniguchi T. Levy-Wilson B. (1997). Characterization of hepatic-specific regulatory elements in the promoter region of the human cholesterol 7alpha-hydroxylase gene. J. Biol. Chem., 272. 9013589

[B7] Crismaru I. Pantea Stoian A. Bratu O. G. Gaman M. A. Stanescu A. M. A. Bacalbasa N. (2020). Low-density lipoprotein cholesterol lowering treatment: The current approach. Lipids Health Dis. 19 (1), 85. 10.1186/s12944-020-01275-x 32375792 PMC7201678

[B8] Deng N. Zhou H. Fan H. Yuan Y. (2017). Single nucleotide polymorphisms and cancer susceptibility. Oncotarget 8 (66), 110635–110649. 10.18632/oncotarget.22372 29299175 PMC5746410

[B9] Donna K Arnett R. S. B. Albert Michelle A. Albert M. A. Buroker A. B. Goldberger Z. D. Hahn E. J. (2019). 2019 ACC/AHA guideline on the primary prevention of cardiovascular disease: A report of the American College of Cardiology/American Heart association task force on clinical practice guidelines. Circulation 140 (11), e596–e646. 10.1161/CIR.0000000000000678 30879355 PMC7734661

[B10] Fu R. H. H. (2015). Change score or followup score? An empirical evaluation of the impact of choice of mean difference estimates rockville (MD). Report No: 15-EHC016-EF. Rockville, Maryland, USA: Agency for Healthcare Research and Quality. 25927135

[B11] Higgins Jpt T. J. Chandler J. Cumpston M. Li T. Page M. J. Welch V. A. (2022). Cochrane handbook for systematic reviews of interventions *version 6.3* . London, England: Cochrane Traning.

[B12] Hofman Mk P. H. Zwinderman A. H. Jukema J. W. (2005). Genetic variation in the rate-limiting enzyme in cholesterol catabolism (cholesterol 7alpha-hydroxylase) influences the progression of atherosclerosis and risk of new clinical events. Clin. Sci. (Lond). 108 (6), 539–545. 10.1042/CS20040339 15707388

[B13] Jiang X. Y. Zhang Q. Chen P. Li S. Y. Zhang N. N. Chen X. D. (2012). CYP7A1 polymorphism influences the LDL cholesterol-lowering response to atorvastatin. J. Clin. Pharm. Ther. 37 (6), 719–723. 10.1111/j.1365-2710.2012.01372.x 22882727

[B14] Kajinami K. Brousseau M. E. Ordovas J. M. Schaefer E. J. (2005). A promoter polymorphism in cholesterol 7alpha-hydroxylase interacts with apolipoprotein E genotype in the LDL-lowering response to atorvastatin. Atherosclerosis 180 (2), 407–415. 10.1016/j.atherosclerosis.2004.12.019 15910869

[B15] Lecerf J. M. de Lorgeril M. (2011). Dietary cholesterol: From physiology to cardiovascular risk. Br. J. Nutr. 106 (1), 6–14. 10.1017/S0007114511000237 21385506

[B16] Li Q. H. J. Wu J. Huang Z. X. Li Q. J. Yin R. X. Lin Q. Z. (2014). The role of common variants of ABCB1 and CYP7A1 genes in serum lipid levels and lipid-lowering efficacy of statin treatment: A meta-analysis. J. Clin. Lipidol. 8 (6), 618–629. 10.1016/j.jacl.2014.07.010 25499945

[B17] Liberati A. Altman D. G. Tetzlaff J. Mulrow C. Gøtzsche P. C. Ioannidis J. P. A. (2009). The PRISMA statement for reporting systematic reviews and meta-analyses of studies that evaluate healthcare interventions: Explanation and elaboration. BMJ 339, b2700. 10.1136/bmj.b2700 19622552 PMC2714672

[B18] Liu Z.-K. Zeng W. Zhang Y. Jia J. Li J. P. Huo Y. (2017). A novel CYP7A1 polymorphism is associated with the low-density lipoprotein cholesterol response to atorvastatin. Vasc. Dis. Ther. 2 (5). 10.15761/vdt.1000135

[B19] Molowa Dt C. W. Cimis G. M. Tan C. P. (1992). Transcriptional regulation of the human cholesterol 7 alpha-hydroxylase gene. Biochemistry 9. 10.1021/bi00124a014 1312351

[B20] Na Liu G. Y. Liu Y. Hu M. Cai Y. Hu Z. Jia C. (2020). Effect of cytochrome P450 7A1 (CYP7A1) polymorphism on lipid responses to simvastatin treatment. J. Cardiovasc Pharmacol. 75, 168–173. 10.1097/FJC.0000000000000774 31663874

[B21] Noyes J. D. Mordi I. R. Doney A. S. Jamal R. Lang C. C. (2021). Precision medicine and adverse drug reactions related to cardiovascular drugs. Diseases 9 (3), 55. 10.3390/diseases9030055 34449608 PMC8396016

[B22] Pandak W. M. Schwarz C. Hylemon P. B. Mallonee D. Valerie K. Heuman D. M. (2001). Effects of CYP7A1 overexpression on cholesterol and bile acid homeostasis. Am. J. Physiology-Gastrointestinal Liver Physiology 281 (4), G878–G889. 10.1152/ajpgi.2001.281.4.G878 11557507

[B23] Parhofer Kg L. U. Laufs U. (2019). The diagnosis and treatment of hypertriglyceridemia. Dtsch. Arztebl Int. 116 (49), 825–832. 10.3238/arztebl.2019.0825 31888796 PMC6962767

[B24] Pullinger C. R. Eng C. Salen G. Shefer S. Batta A. K. Erickson S. K. (2002). Human cholesterol 7alpha-hydroxylase (CYP7A1) deficiency has a hypercholesterolemic phenotype. J. Clin. Investigation 110 (1), 109–117. 10.1172/JCI15387 PMC151029 12093894

[B25] RobertOh C. J. B. L. (2007). Management of hypertriglyceridemia. Am. Fam. Physician 75 (9), 1365–1371. 17508532

[B26] Schachter M. (2004). Chemical, pharmacokinetic and pharmacodynamic properties of statins: An update. Fundam. Clin. Pharmacol. 19, 117–125. 10.1111/j.1472-8206.2004.00299.x 15660968

[B27] Scott M. G. Stone N. J. Bailey A. L. Beam C. Birtcher K. K. Blumenthal R. S. (2018). 2018 AHA/ACC/AACVPR/AAPA/ABC/ACPM/ADA/AGS/APhA/ASPC/NLA/PCNA guideline on the management of blood cholesterol: Executive summary. J. Am. Coll. Cardiol. 73 (24), 3168–3209. 10.1016/j.jacc.2018.11.002 30423391

[B28] Shah R. R. Smith R. L. (2015). Addressing phenoconversion: The achilles' heel of personalized medicine. Br. J. Clin. Pharmacol. 79 (2), 222–240. 10.1111/bcp.12441 24913012 PMC4309629

[B29] Wang D. Hartmann K. Seweryn M. Sadee W. (2018). Interactions between regulatory variants in CYP7A1 (cholesterol 7α-hydroxylase) promoter and enhancer regions regulate CYP7A1 expression. Circ. Genom Precis. Med. 11 (10), e002082. 10.1161/CIRCGEN.118.002082 30354296 PMC6211808

[B30] Wei K.-K. M. D. Zhang L.-R. Zhang Y. M. D. Hu X.-J. M. D. (2011). Interactions between CYP7A1 A-204C and ABCG8 C1199A polymorphisms on lipid lowering with atorvastatin. J. Clin. Pharm. Ther. 36 (6), 725–733. 10.1111/j.1365-2710.2010.01227.x 21128988

