# Supplementary material for: A meta-analysis of the pooled impact of CYP7A1 single nucleotide polymorphisms on serum lipid responses to statins
Source: Front Genet. 2023 Jun 9;14:1199549. doi: 10.3389/fgene.2023.1199549 (PMC10292746; doi:10.3389/fgene.2023.1199549)
Supplement: Supplementary file 1 [file Table1.DOCX]

**Supplementary Materials**

Supplementary Table 1 *Literature search strategy used for electronic databases (PUBMED)*

| 1 | Single nucleotide polymorphism | "Genetic Variation"[Mesh] OR "Genetic Variation" OR "Genetic Variations" OR "Variations, Genetic" OR "Variation, Genetic" OR "Diversity, Genetic" OR "Diversities, Genetic" OR "Genetic Diversities" OR "Genetic Diversity" OR "Geneti* Divers*" |
| --- | --- | --- |
| 2 | CYP7A1 | "Cholesterol 7-alpha-Hydroxylase"[Mesh] OR "Cholesterol 7-alpha-Hydroxylase" OR "Cholesterol 7 alpha Hydroxylase" OR "Cholesterol 7alpha-Hydroxylase" OR "Cholesterol 7alpha Hydroxylase" OR "Cholesterol-7-Hydroxylase" OR "Cholesterol 7 Hydroxylase" OR "Cytochrome P-450 CYP7" OR "Cytochrome P 450 CYP7" OR "CYP7A" OR "Cholesterol 7-alpha-Monooxygenase" OR "Cholesterol 7 alpha Monooxygenase" OR "Cytochrome P450 7" OR "CYP7" OR "CYP 7A" OR "CYP 7" OR "CYP7A1 protein, human"[Mesh] OR "CYP7 protein, human" OR "cholesterol-7-alpha-hydroxylase, human" OR "cytochrome P450, family 7, subfamily A, polypeptide 1, human" OR "cytochrome P450, subfamily VIIA (cholesterol 7 alpha-monooxygenase), polypeptide 1, human" |
| 3 | Statins | "Hydroxymethylglutaryl-CoA Reductase Inhibitors"[Mesh] OR "Hydroxymethylglutaryl-CoA Reductase Inhibitors" OR "Hydroxymethylglutaryl CoA Reductase Inhibitors" OR "Inhibitors, Hydroxymethylglutaryl-CoA Reductase" OR "Reductase Inhibitors, Hydroxymethylglutaryal-CoA" OR "HMG-CoA Reductase Inhibitor" OR "HMG CoA Reductase Inhibitor" OR "Statin" OR "Statins, HMG-CoA" OR "HMG-CoA Statins" OR "Statins, HMG CoA" OR "Inhibitors, HMG-CoA Reductase" OR "Inhibitors, HMG CoA Reductase" OR "Reductase Inhibitors, HMG-CoA" OR "HMG-CoA Reductase Inhibitors" OR "HMG CoA Reductase Inhibitors" OR "Inhibitors, Hydroxymethylglutaryl-Coenzyme A" OR "Hydroxymethylglutaryl-Coenzyme A Inhibitors" OR "Inhibitors, Hydroxymethylglutaryl Coenzyme A" OR "Inhibitors, Hydroxymethylglutaryl-CoA" OR "Hydroxymethylglutaryl-CoA Inhibitors" OR "Inhibitors, Hydroxymethylglutaryl CoA" OR "Hydroxymethylglutaryl-CoA Reductase Inhibitor" OR "Hydroxymethylglutaryl CoA Reductase Inhibitor" OR "Reductase Inhibitor, Hydroxymethylglutaryl-CoA" OR "Statins" |
| 4 | Atorvastatin | "Atorvastatin"[Mesh] OR "Atorvastatin" OR "(3R,5R)-7-(2-(4-Fluorophenyl)-5-isopropyl-3-phenyl-4-(phenylcarbamoyl)-1H-pyrrol-1-yl)-3,5-dihydroxyheptanoic acid" OR "Atorvastatin Calcium" OR "Atorvastatin, Calcium Salt" OR "Liptonorm" OR "Lipitor" OR "Atorvastatin Calcium Hydrate" OR "Atorvastatin Calcium Anhydrous" OR "CI 981" OR "CI-981" OR "CI981" OR "Atorvastatin Calcium Trihydrate" |
| 5 | Simvastatin | "Simvastatin"[Mesh] OR "Simvastatin" OR "Zocor" OR "MK-733" OR "MK 733" OR "MK733" OR "Synvinolin" |
| 6 | Lovastatin | "Lovastatin"[Mesh] OR "Lovastatin" OR "Mevinolin" OR "Monacolin K" OR "6-Methylcompactin" OR "6 Methylcompactin" OR "MK-803" OR "MK 803" OR "MK803" OR "Mevacor" OR "Lovastatin, (1 alpha(S*))-Isomer" OR "Lovastatin, 1 alpha-Isomer" OR "1 alpha-Isomer Lovastatin" OR "Lovastatin, 1 alpha Isomer" OR "alpha-Isomer Lovastatin, 1" |
| 7 | Pravastatin | "Pravastatin"[Mesh] OR "Pravastatin" OR "Eptastatin" OR "Vasten" OR "CS-514" OR "CS 514" OR "CS514" OR "Lin-Pravastatin" OR "Lin Pravastatin" OR "Lipemol" OR "Liplat" OR "Nu-Pravastatin" OR "Nu Pravastatin" OR "Prareduct" OR "Mevalotin" OR "Pravachol" OR "Elisor" OR "Selektine" OR "Lipostat" OR "Pravacol" OR "Pravasin" OR "Pravastatin Monosodium Salt, (6 beta)-Isomer" OR "Pravastatin Sodium" OR "Pravastatin Sodium Salt" OR "Sodium Salt, Pravastatin" OR "Pravastatin tert-Octylamine Salt" OR "Pravastatin tert Octylamine Salt" OR "Pravastatin, (6 beta)-Isomer" OR "RMS-431" OR "RMS 431" OR "RMS431" OR "SQ-31000" OR "SQ 31000" OR "SQ31000" OR "SQ-31,000" OR "SQ 31,000" OR "SQ31,000" OR "Apo-Pravastatin" OR "Apo Pravastatin" OR "Bristacol" |
| 8 | Rosuvastatin | "Rosuvastatin Calcium"[Mesh] OR "Rosuvastatin Calcium" OR "Calcium, Rosuvastatin" OR "Crestor" OR "Rosuvastatin" OR "ZD4522" OR "ZD 4522" |
| 9 | Pitavastatin | "pitavastatin"[Mesh] OR "pitavastatin" OR "itavastatin" OR "(E,3R,5S)-7-(2-cyclopropyl-4-(4-fluorophenyl)quinolin-3-yl)-3,5-dihydroxyhept-6-enoic acid" OR "P 872441" OR "P-872441" OR "NK 104" OR "NK-104" OR "pitavastatin calcium" OR "itavastatin calcium" OR "pitavastatin lactone" OR "nisvastatin" |
| 10 | Fluvastatin | "Fluvastatin"[Mesh] OR "Fluvastatin" OR "Fluvastatin Sodium" OR "Fluvastatin Sodium Salt" OR "Fluindostatin" OR "Lescol" OR "XU 62-320" OR "XU 62 320" OR "XU-62320" OR "XU62320" OR "XU 62320" OR "7-(3-(4-Fluorophenyl)-1-(1-methylethyl)-1H-indol-2-yl)-3,5-dihydroxy-6-heptenoate" |
| 11 | Cerivastatin | "cerivastatin"[Mesh] OR "cerivastatin" OR "Kazak" OR "cerivastatin sodium" OR "6-Heptenoic acid, 7-(4-(4-fluorophenyl)-5-(methoxymethyl)-2,6-bis(1-methylethyl)-3-pyridinyl)-3,5-dihydroxy-, monosodium salt, (S-(R*,S*-(E)))-" OR "7-(4-(4-fluorophenyl)-2,6-diisopropyl-5-(methoxymethyl)pyrid-3-yl)-3,5-dihydroxy-6-heptenoate sodium salt" OR "rivastatin" OR "Certa" OR "Bay w 6228" OR "Baycol" OR "Lipobay" |
| Searches performed | #1 AND #2 AND (#3 to #11, connector OR) | |

Supplementary Table 2 *Literature search strategy used for electronic databases (Cochrane)*

| 1 | CYP7A1 | [mh "Cholesterol 7-alpha-Hydroxylase"] OR "Cholesterol 7-alpha-Hydroxylase" OR "Cholesterol 7 alpha Hydroxylase" OR "Cholesterol 7alpha-Hydroxylase" OR "Cholesterol 7alpha Hydroxylase" OR "Cholesterol-7-Hydroxylase" OR "Cholesterol 7 Hydroxylase" OR "Cytochrome P-450 CYP7" OR "Cytochrome P 450 CYP7" OR "CYP7A" OR "Cholesterol 7-alpha-Monooxygenase" OR "Cholesterol 7 alpha Monooxygenase" OR "Cytochrome P450 7" OR "CYP7" OR "CYP 7A" OR "CYP 7" OR "CYP7A1" |
| --- | --- | --- |
| 2 | Genetic Variation | [mh "Genetic Variation"] OR "Genetic Variation" OR "Genetic Variations" OR "Variations, Genetic" OR "Variation, Genetic" OR "Diversity, Genetic" OR "Diversities, Genetic" OR "Genetic Diversities" OR "Genetic Diversity" |
| 3 | Allele | [mh "Allele"] OR "Allele" OR "Allelomorphs" OR "Allelomorph" |
| 4 | Statins | [mh "Hydroxymethylglutaryl-CoA Reductase Inhibitors"] OR "Hydroxymethylglutaryl-CoA Reductase Inhibitors" OR "Hydroxymethylglutaryl CoA Reductase Inhibitors" OR "Inhibitors, Hydroxymethylglutaryl-CoA Reductase" OR "Reductase Inhibitors, Hydroxymethylglutaryl-CoA" OR "HMG-CoA Reductase Inhibitor" OR "HMG CoA Reductase Inhibitor" OR "Statin" OR "Statins, HMG-CoA" OR "HMG-CoA Statins" OR "Statins, HMG CoA" OR "Inhibitors, HMG-CoA Reductase" OR "Inhibitors, HMG CoA Reductase" OR "Reductase Inhibitors, HMG-CoA" OR "HMG-CoA Reductase Inhibitors" OR "HMG CoA Reductase Inhibitors" OR "Inhibitors, Hydroxymethylglutaryl-Coenzyme A" OR "Hydroxymethylglutaryl-Coenzyme A Inhibitors" OR "Inhibitors, Hydroxymethylglutaryl Coenzyme A" OR "Inhibitors, Hydroxymethylglutaryl-CoA" OR "Hydroxymethylglutaryl-CoA Inhibitors" OR "Inhibitors, Hydroxymethylglutaryl CoA" OR "Hydroxymethylglutaryl-CoA Reductase Inhibitor" OR "Hydroxymethylglutaryl CoA Reductase Inhibitor" OR "Reductase Inhibitor, Hydroxymethylglutaryl-CoA" OR "Statins" |
| 5 | Atorvastatin | [mh "Atorvastatin"] OR "Atorvastatin" OR "(3R,5R)-7-(2-(4-Fluorophenyl)-5-isopropyl-3-phenyl-4-(phenylcarbamoyl)-1H-pyrrol-1-yl)-3,5-dihydroxyheptanoic acid" OR "Atorvastatin Calcium" OR "Atorvastatin, Calcium Salt" OR "Liptonorm" OR "Lipitor" OR "Atorvastatin Calcium Hydrate" OR "Atorvastatin Calcium Anhydrous" OR "CI 981" OR "CI-981" OR "CI981" OR "Atorvastatin Calcium Trihydrate" |
| 6 | Simvastatin | [mh "Simvastatin"] OR "Simvastatin" OR "Zocor" OR "MK-733" OR "MK 733" OR "MK733" OR "Synvinolin" |
| 7 | Lovastatin | [mh "Lovastatin"] OR "Lovastatin" OR "Mevinolin" OR "Monacolin K" OR "6-Methylcompactin" OR "6 Methylcompactin" OR "MK-803" OR "MK 803" OR "MK803" OR "Mevacor" OR "Lovastatin, (1 alpha(S*))-Isomer" OR "Lovastatin, 1 alpha-Isomer" OR "1 alpha-Isomer Lovastatin" OR "Lovastatin, 1 alpha Isomer" OR "alpha-Isomer Lovastatin, 1" |
| 8 | Pravastatin | [mh "Pravastatin"] OR "Pravastatin" OR "Eptastatin" OR "Vasten" OR "CS-514" OR "CS 514" OR "CS514" OR "Lin-Pravastatin" OR "Lin Pravastatin" OR "Lipemol" OR "Liplat" OR "Nu-Pravastatin" OR "Nu Pravastatin" OR "Prareduct" OR "Mevalotin" OR "Pravachol" OR "Elisor" OR "Selektine" OR "Lipostat" OR "Pravacol" OR "Pravasin" OR "Pravastatin Monosodium Salt, (6 beta)-Isomer" OR "Pravastatin Sodium" OR "Pravastatin Sodium Salt" OR "Sodium Salt, Pravastatin" OR "Pravastatin tert-Octylamine Salt" OR "Pravastatin tert Octylamine Salt" OR "Pravastatin, (6 beta)-Isomer" OR "RMS-431" OR "RMS 431" OR "RMS431" OR "SQ-31000" OR "SQ 31000" OR "SQ31000" OR "SQ-31,000" OR "SQ 31,000" OR "SQ31,000" OR "Apo-Pravastatin" OR "Apo Pravastatin" OR "Bristacol" |
| 9 | Rosuvastatin | [mh "Rosuvastatin Calcium"] OR "Rosuvastatin Calcium" OR "Calcium, Rosuvastatin" OR "Crestor" OR "Rosuvastatin" OR "ZD4522" OR "ZD 4522" |
| 10 | Pitavastatin | [mh "pitavastatin"] OR "pitavastatin" OR "itavastatin" OR "(E,3R,5S)-7-(2-cyclopropyl-4-(4-fluorophenyl)quinolin-3-yl)-3,5-dihydroxyhept-6-enoic acid" OR "P 872441" OR "P-872441" OR "NK 104" OR "NK-104" OR "pitavastatin calcium" OR "itavastatin calcium" OR "pitavastatin lactone" OR "nisvastatin" |
| 11 | Fluvastatin | [mh "Fluvastatin"] OR "Fluvastatin" OR "Fluvastatin Sodium" OR "Fluvastatin Sodium Salt" OR "Fluindostatin" OR "Lescol" OR "XU 62-320" OR "XU 62 320" OR "XU-62320" OR "XU62320" OR "XU 62320" OR "7-(3-(4-Fluorophenyl)-1-(1-methylethyl)-1H-indol-2-yl)-3,5-dihydroxy-6-heptenoate" |
| 12 | Cerivastatin | [mh "cerivastatin"] OR "cerivastatin" OR "Kazak" OR "cerivastatin sodium" OR "6-Heptenoic acid, 7-(4-(4-fluorophenyl)-5-(methoxymethyl)-2,6-bis(1-methylethyl)-3-pyridinyl)-3,5-dihydroxy-, monosodium salt, (S-(R*,S*-(E)))-" OR "7-(4-(4-fluorophenyl)-2,6-diisopropyl-5-(methoxymethyl)pyrid-3-yl)-3,5-dihydroxy-6-heptenoate sodium salt" OR "rivastatin" OR "Certa" OR "Bay w 6228" OR "Baycol" OR "Lipobay" |
| Searches performed | (#1 AND #2 AND #4) AND (#4 to #12; connector OR) | |

Supplementary Table 3 *Literature search strategy used for electronic databases (EMBASE)*

| 1 | CYP7A1 | 'Cholesterol 7alpha monooxygenase'/exp OR 'Cholesterol 7 alpha hydroxylase' OR 'cholesterol 7 alpha monooxygenase' OR 'cholesterol 7 alpha-monooxygenase' OR 'cholesterol 7-alpha-hydroxylase' OR 'cholesterol 7alpha hydroxylase' OR 'cholesterol, nadph:oxygen oxidoreductase (7alpha hydroxylating)' OR 'cholesterol-7-hydroxylase' OR 'CYP7A1' OR 'cytochrome P450 7A1' OR 'cytochrome P450 CYP7A1' OR 'e.c. 1.14.13.17' |
| --- | --- | --- |
| 2 | Single Nucleotide Polymorphism | 'Single nucleotide polymorphism'/exp OR 'polymorphism*, single nucleotide' OR 'single nucleotide variant*' OR 'single nucleotide variation*' or 'SNP*" |
| 3 | Statins | 'Hydroxymethylglutaryl - CoA Reductase Inhibitors'/exp OR 'HMG CoA reductase inhibitor' OR 'HMG CoA reductase inhibitors' OR 'hmg coenzyme a reductase inhibitor' OR 'hmg-coa reductase inhibitors' OR 'hydroxymethylglutaryl coa reductase inhibitors' OR 'hydroxymethylglutaryl-coa reductase inhibitors' OR 'statin (drug)' OR 'statins' OR 'vastatin' |
| 4 | Lipoproteins | 'Lipoprotein'/exp OR 'lipopolypeptide*' OR 'lipoprotein complex*' OR 'lipoprotein polypeptide*' OR 'lipoprotein*' |
| 5 | Triglycerides | 'Triacylglycerol'/exp OR 'acylglycerol*, tri' OR 'fatty acid triglyceride*' OR 'triacyl glyceride*' OR 'triglyceride' OR 'triglycerides' OR 'tryglyceride*' |
| 6 | Cholesterol | 'Cholesterol'/exp OR '3 hydroxy 5 cholestene' OR '3beta hydroxy 5 cholestene' OR '3beta hydroxycholest 5 ene' OR '5 cholesten 3beta ol' OR 'beta cholesterol' OR 'cholest 5 en 3beta ol' OR 'cholest 5 ene 3 ol' OR 'cholesterin' OR 'cholesterine' OR 'cholesterol release' OR 'dythol' OR 'nsc 8798' |
| Searches performed | #1 AND #2 AND #3 AND (#4 OR #5 OR #6) | |

Supplementary Table 4 *High-, Moderate- and Low-Intensity Statin Therapy*

| High Intensity  (LDL-C lowering ≥50%) | Moderate Intensity  (LDL-C lowering 30%-49%) | Low Intensity  (LDL-C lowering <30%) |
| --- | --- | --- |
| Atorvastatin 40 mg (80 mg)  Rosuvastatin 20 mg (40 mg) | Atorvastatin 10 mg (20 mg)  Rosuvastatin (5 mg) 10 mg  Simvastatin 20-40 mg  Pravastatin 40 mg (80 mg)  Lovastatin 40 mg (80 mg)  Fluvastatin Extended Release 80 mg  Fluvastatin 40 mg twice daily  Pitavastatin 1-4 mg | Simvastatin 10 mg  Pravastatin 10-20 mg  Lovastatin 20 mg  Fluvastatin 20-40 mg |

*Doses in brackets are approved by the Food and Drug Administration (FDA) but not proven in trials*


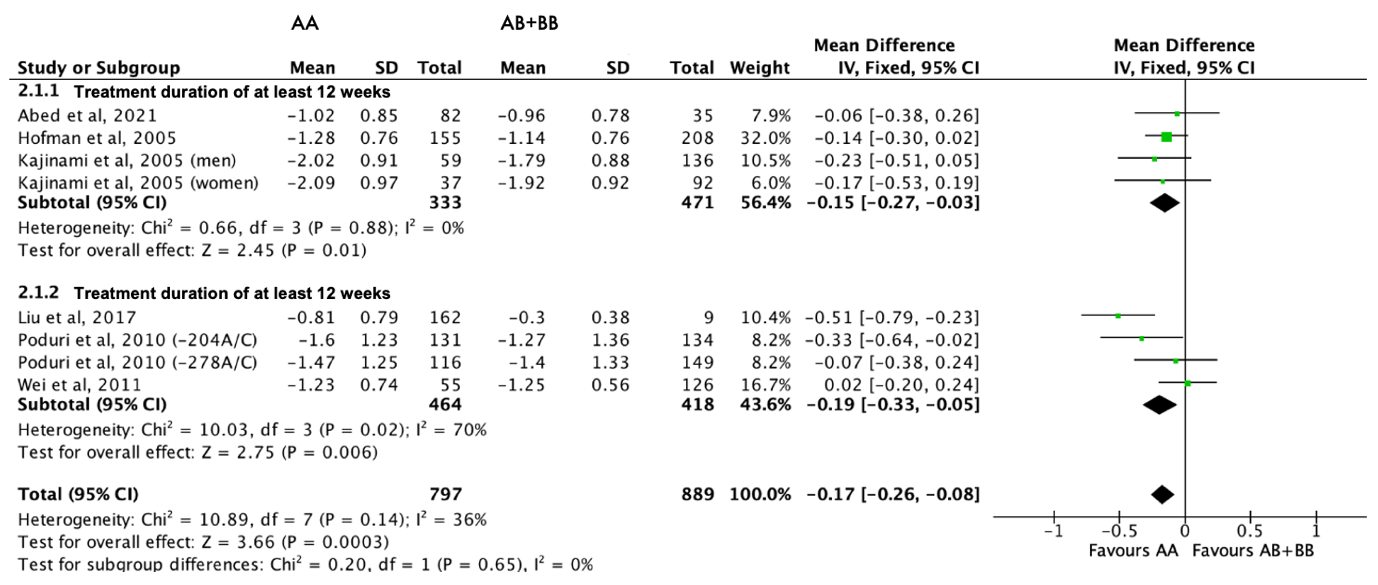


Supplementary Figure 1 Mean difference in total cholesterol lowering response to statins between carriers (AB+BB) versus non-carriers (AA) of CYP7A1 SNPs using a random-effects model, stratified by treatment duration: r=0


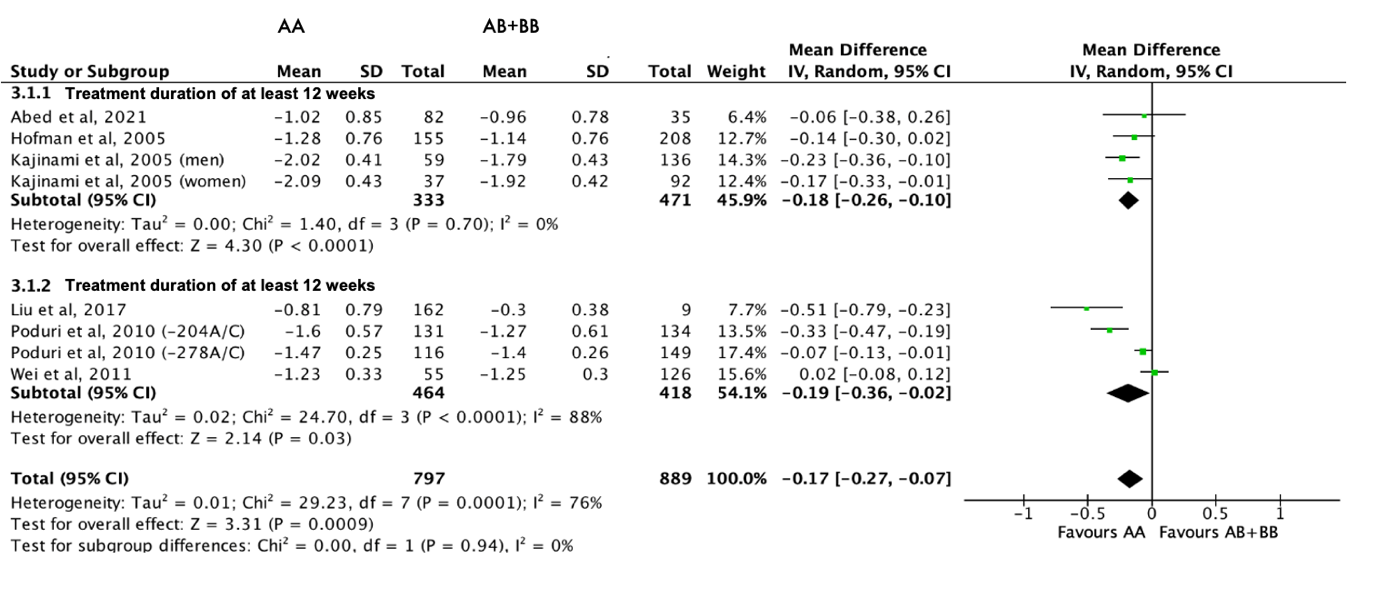


Supplementary Figure 2 Mean difference in total cholesterol lowering response to statins between carriers (AB+BB) versus non-carriers (AA) of CYP7A1 SNPs using a random-effects model, stratified by treatment duration: r=0.8


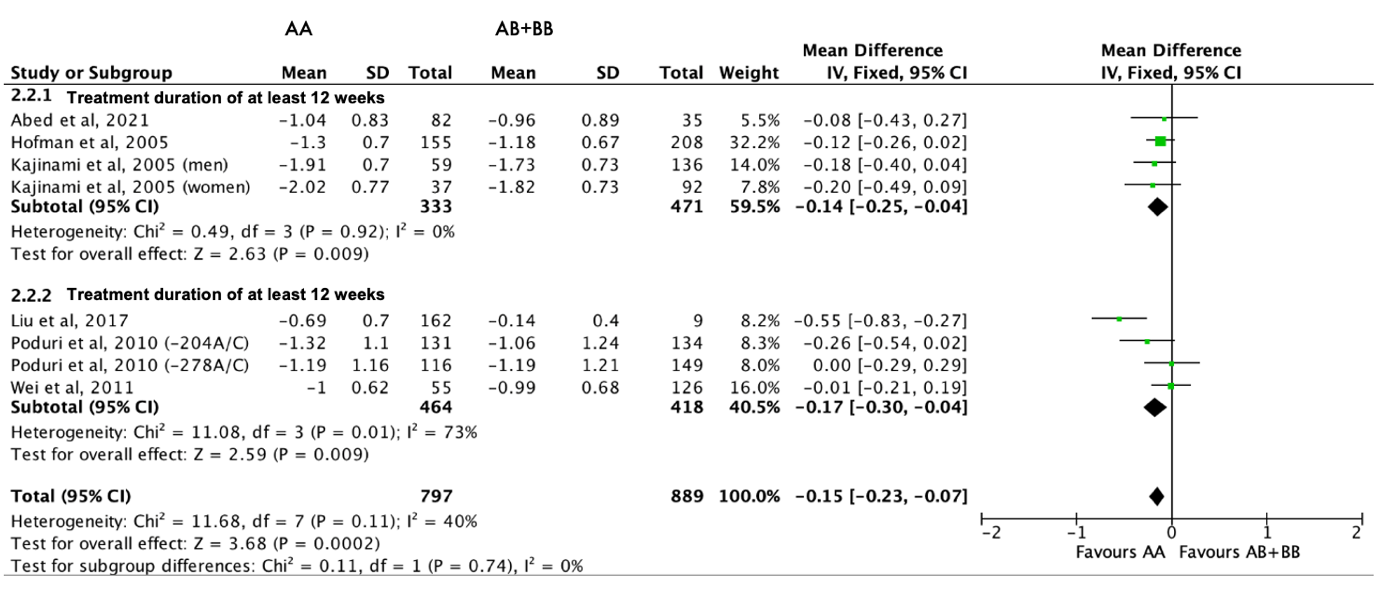


Supplementary Figure 3 Mean difference in LDL-C lowering response to statins between carriers (AB+BB) versus non-carriers (AA) of CYP7A1 SNPs using a fixed-effects model, stratified by treatment duration: r=0


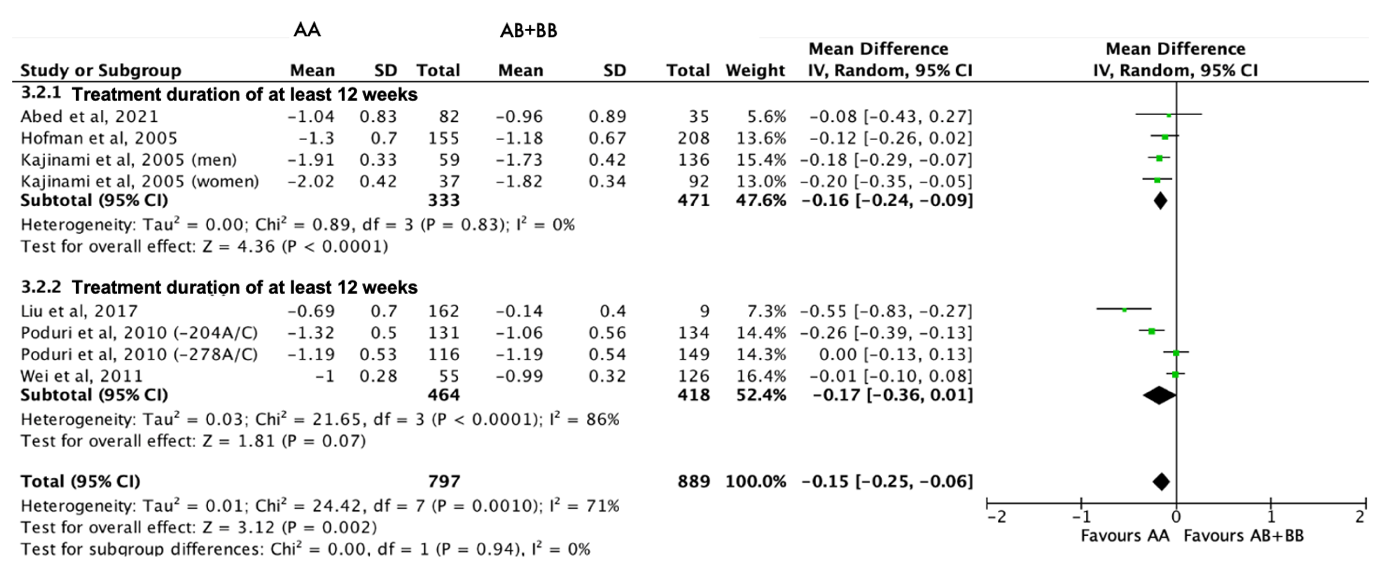


Supplementary Figure 4 Mean difference in LDL-C lowering response to statins between carriers (AB+BB) versus non-carriers (AA) of CYP7A1 SNPs using a fixed-effects model, stratified by treatment duration: r=0.8


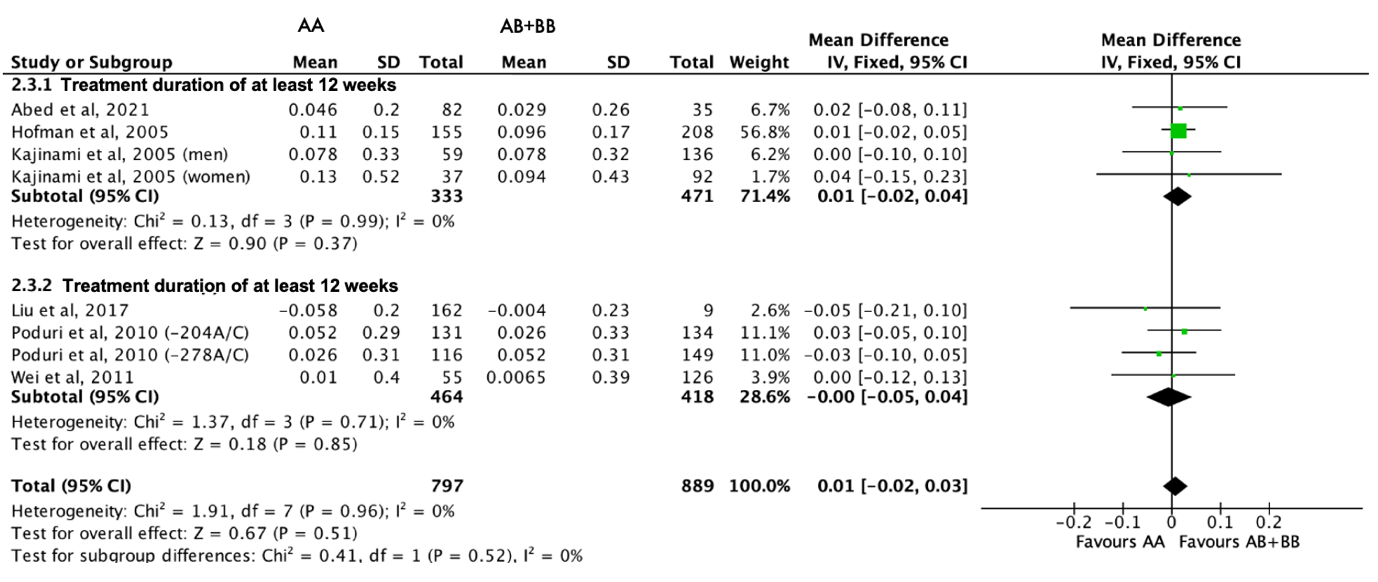


Supplementary Figure 5 Mean difference in HDL-C improvement response to statins between carriers (AB+BB) versus non-carriers (AA) of CYP7A1 SNPs using a fixed-effects model, stratified by treatment duration: r=0


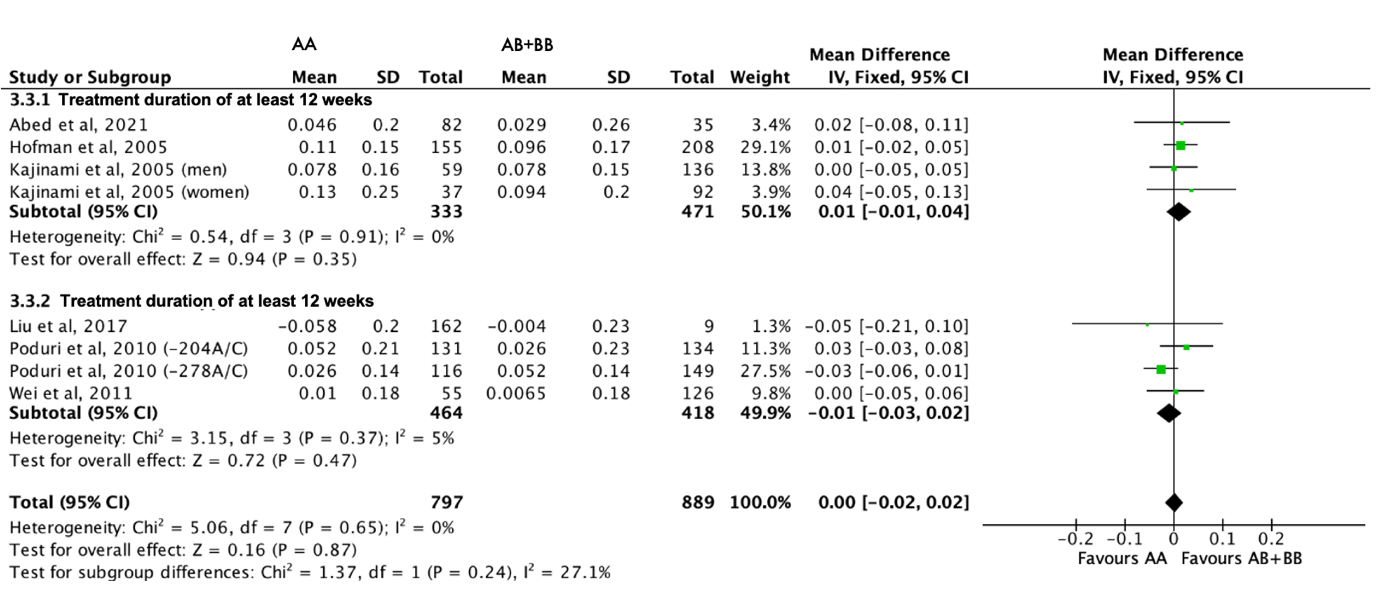


Supplementary Figure 6 Mean difference in HDL-C improvement response to statins between carriers (AB+BB) versus non-carriers (AA) of CYP7A1 SNPs using a fixed-effects model, stratified by treatment duration: r=0.8


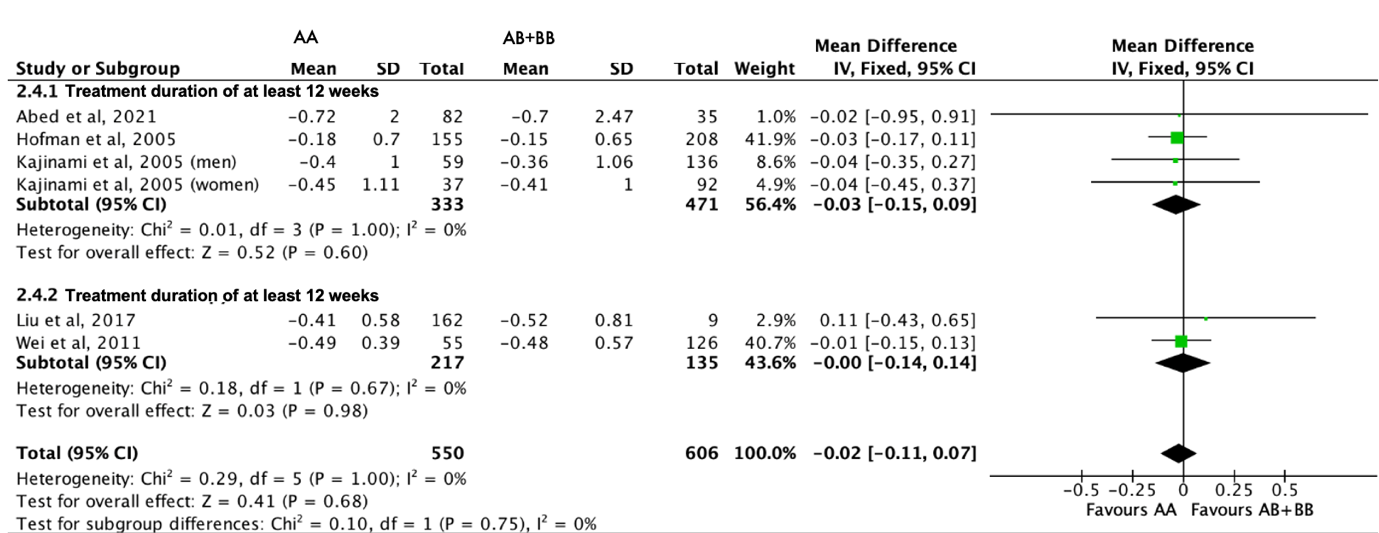


Supplementary Figure 7 Mean difference in triglycerides lowering response to statins between carriers (AB+BB) versus non-carriers (AA) of CYP7A1 SNPs using a fixed-effects model, stratified by treatment duration: r=0


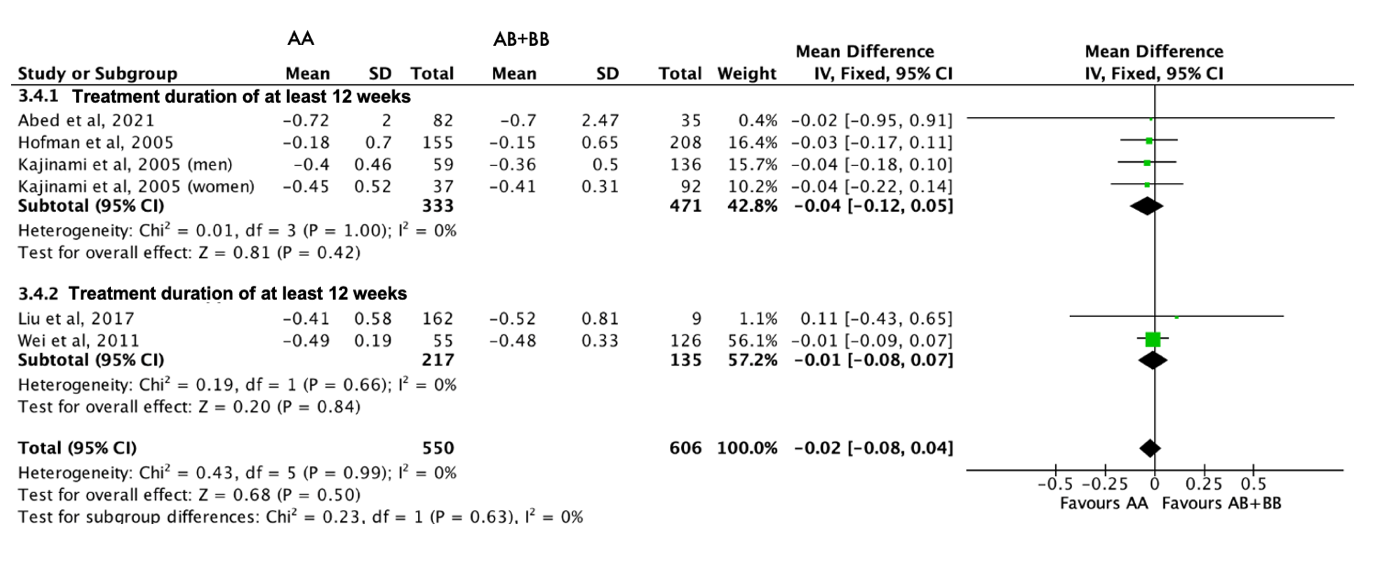


Supplementary Figure 8 Mean difference in triglycerides lowering response to statins between carriers (AB+BB) versus non-carriers (AA) of CYP7A1 SNPs using a fixed-effects model, stratified by treatment duration: r=0.8
